# Limitations of the Impagliazzo–Nisan–Wigderson Pseudorandom Generator Against Permutation Branching Programs

**DOI:** 10.1007/s00453-024-01251-2

**Published:** 2024-07-29

**Authors:** William M. Hoza, Edward Pyne, Salil Vadhan

**Affiliations:** 1 University of Chicago, Chicago, USA; 2 MIT, Cambridge, USA; 3 Harvard University, Cambridge, USA

**Keywords:** Pseudorandomness, Space-bounded computation, Spectral graph theory

## Abstract

The classic Impagliazzo–Nisan–Wigderson (INW) pseudorandom generator (PRG) (STOC ‘94) for space-bounded computation uses a seed of length $O(\log n \cdot \log (nw/\varepsilon )+\log d)$ to fool ordered branching programs of length *n*, width *w*, and alphabet size *d* to within error $\varepsilon$. A series of works have shown that the analysis of the INW generator can be improved for the class of *permutation* branching programs or the more general *regular* branching programs, improving the $O(\log ^2 n)$ dependence on the length *n* to $O(\log n)$ or ${\tilde{O}}(\log n)$. However, when also considering the dependence on the other parameters, these analyses still fall short of the optimal PRG seed length $O(\log (nwd/\varepsilon ))$. In this paper, we prove that any “spectral analysis” of the INW generator requires seed length

$$\begin{aligned} \Omega \left( \log n\cdot \log \log \left( \min \{n,d\}\right) +\log n\cdot \log \left( w/\varepsilon \right) +\log d\right) \end{aligned}$$

to fool ordered permutation branching programs of length *n*, width *w*, and alphabet size *d* to within error $\varepsilon$. By “spectral analysis” we mean an analysis of the INW generator that relies only on the spectral expansion of the graphs used to construct the generator; this encompasses all prior analyses of the INW generator. Our lower bound matches the upper bound of Braverman–Rao–Raz–Yehudayoff (FOCS 2010, SICOMP 2014) for regular branching programs of alphabet size $d=2$ except for a gap between their $O\left( \log n \cdot \log \log n\right)$ term and our $\Omega \left( \log n \cdot \log \log \min \{n,d\}\right)$ term. It also matches the upper bounds of Koucký–Nimbhorkar–Pudlák (STOC 2011), De (CCC 2011), and Steinke (ECCC 2012) for constant-width ($w=O(1)$) permutation branching programs of alphabet size $d=2$ to within a constant factor. To fool permutation branching programs in the measure of *spectral norm*, we prove that any spectral analysis of the INW generator requires a seed of length $\Omega \left( \log n\cdot \log \log n+\log n\cdot \log (1/\varepsilon )\right)$ when the width is at least polynomial in *n* ($w=n^{\Omega (1)}$), matching the recent upper bound of Hoza–Pyne–Vadhan (ITCS 2021) to within a constant factor.

## Introduction

Starting with the work of Babai et al. [2], there has been three decades of work of constructing and analyzing pseudorandom generators for space-bounded computation, motivated by obtaining unconditional derandomization (e.g. seeking to prove that $\textrm{BPL}=\textrm{L}$) and a variety of other applications (e.g. [3–6]). Although we still remain quite far from having pseudorandom generators that suffice for a full derandomization of space-bounded computation, there has been substantial progress on pseudorandom generators for restricted models of space-bounded computation. In particular, a series of works has shown that the analysis of the classic Impagliazzo–Nisan–Wigderson (INW) generator [7] can be significantly improved for restricted models (e.g. “permutation branching programs”), but these analyses have not matched the parameters of an optimal pseudorandom generator. In this work, we show that there are inherent limitations to the analysis of the INW generator for these restricted models, proving lower bounds that nearly match the known upper bounds.

### Pseudorandom Generators for Space-Bounded Computation

Like previous work, we will work with the following nonuniform model of space-bounded computation.

#### Definition 1.1

An *ordered branching program* *B* of *length* *n*, *width* *w* and *alphabet size* *d* computes a function $B:{[d]^n}\rightarrow \{0,1\}$. On an input $\sigma \in {[d]^n}$, the branching program computes as follows. It starts at a fixed start state $v_0\in [w]$. Then for $t=1,\ldots ,n$, it reads the next symbol $\sigma _t$ and updates its state according to a transition function $B_t:[w]\times [d]\rightarrow [w]$ by taking $v_{t}=B_t(v_{t-1},\sigma _t)$. Note that the transition function $B_t$ can differ at each time step.

Moreover, there is a set of accept states $V_e \subseteq [w]$. Let *u* be the final state of the branching program on input $\sigma$. If $u\in V_e$ the branching program accepts, denoted $B(\sigma )=1$, and otherwise the program rejects, denoted $B(\sigma )=0$.

An ordered branching program can be viewed as a layered digraph, consisting of $n+1$ layers of *w* vertices each, where for every $t=1,\ldots ,n$ and $v\in [w]$, the *v*’th vertex in layer $t-1$ has *d* outgoing edges, going to the vertices $B_t(v,1),B_t(v,2),\ldots ,B_t(v,d)\in [w]$ in layer *t*.

An ordered branching program corresponds to a streaming algorithm, in that the *n* input symbols from [*d*] are each read only once, and in a fixed order. This is the relevant model for derandomization of space-bounded computation because a randomized space-bounded algorithm processes its random bits in a streaming fashion. Specifically, if on an input *x*, a randomized Turing machine *A* uses space *s* and *n* random bits $\sigma$, the function $B_x(\sigma ) = A(x;\sigma )$ can be computed by an ordered branching program of length *n*, width $w=2^{s+O(\log s)}\cdot O(|x|)$ and alphabet size 2. In particular, if *A* is a randomized logspace algorithm (i.e. a BPL algorithm), then $n=w=\text {poly}(|x|)$.

The standard definition of pseudorandom generator is as follows.

#### Definition 1.2

Let ${\mathcal {F}}$ be a class of functions $f: {[d]^n}\rightarrow \{0, 1\}$. An $\varepsilon$-*pseudorandom generator* ($\varepsilon$-*PRG*) for ${\mathcal {F}}$ is a function ${{\,\textrm{GEN}\,}}: [S] \rightarrow {[d]^n}$ such that for every $f \in {\mathcal {F}}$,

$$\begin{aligned} \left| \mathop {\mathrm {\mathbb {E}}}\limits _{x \leftarrow U_{[d]^n}}[f(x)] - \mathop {\mathrm {\mathbb {E}}}\limits _{x \leftarrow U_{[S]}}[f({{\,\textrm{GEN}\,}}(x))]\right| \le \varepsilon , \end{aligned}$$

where $U_{[S]}$ is the uniform distribution over the set $[S]=\{0,\ldots ,S - 1\}$. We say $s:=\log (S)$ is the *seed length* of the PRG. We say a generator ${{\,\textrm{GEN}\,}}$ is *explicit* if the *i*th symbol of output is computable in space *O*(*s*). We say that ${{\,\textrm{GEN}\,}}$ $\varepsilon$-*fools* ${\mathcal {F}}$ if it is an $\varepsilon$-PRG for ${\mathcal {F}}$.

By the probabilistic method, it can be shown that there exist (non-explicit) $\varepsilon$-PRGs for the class of ordered branching programs of length *n*, width *w*, and alphabet size *d* with seed length $s=O(\log (nwd/\varepsilon ))$, and it can be shown that this is optimal up to a constant factor (provided that $2^n\ge w$, $n,d,w\ge 2$, and $\varepsilon \le 1/3$). An explicit construction with such a seed length (even for $d=2$ and $\varepsilon =1/3$) would suffice to fully derandomize logspace computation (i.e. prove $\textrm{BPL}=\textrm{L}$).

The classic construction of Impagliazzo, Nisan, and Wigderson [7] gives an explicit PRG with seed length $s = O\left( \log n \cdot \log (nw/\varepsilon ) + \log d\right)$, an improvement over Nisan’s earlier construction [8] in terms of the dependence on *d*. For the case corresponding to derandomizing general logspace computation, where *d* and $\varepsilon$ are constant and *w* is polynomially related to *n*, we have $s=O\left( \log ^2 n\right)$, quadratically worse than the optimal seed length of $s=O(\log n)$. Brody and Verbin [9] showed that these classic pseudorandom generators require seed length $\Omega \left( \log ^2 n\right)$ even for width $w=3$ (see Appendix A). Meka et al. [10] recently gave a completely different explicit construction of pseudorandom generator for width $w=3$ with seed length $s={\tilde{O}}\left( \log n \cdot \log (1/\varepsilon )\right)$, but for width $w=4$ no explicit constructions with seed length $o\left( \log ^2 n\right)$ are known.

### Permutation Branching Programs

Motivated by the lack of progress on the general ordered branching program model, there has been extensive research on restricted models:

#### Definition 1.3

An *(ordered) regular branching program* of length *n*, width *w*, and alphabet size *d* is an ordered branching program where the associated layered digraph consists of regular bipartite graphs between every pair of consecutive layers. Equivalently, for every $t=1,\ldots ,n$ and every $v\in [w]$, there are exactly *d* pairs $(u,\sigma )\in [w]\times [d]$ such that $B_t(u,\sigma )=v$.

#### Definition 1.4

An *(ordered) permutation branching program* of length *n*, width *w*, and alphabet size *d* is an ordered branching program where for all $t \in [n]$ and $\sigma \in [d]$, $B_t(\cdot ,\sigma )$ is a permutation on [*w*].

Every permutation branching program is a regular branching program, but not conversely.

A series of works has shown that the Impagliazzo–Nisan–Wigderson (INW) pseudorandom generator can be instantiated with smaller seed length for regular or permutation branching programs. First, Rozenman and Vadhan [11] analyzed the INW generator for carrying out random walks on *d*-regular *w*-vertex graphs, which correspond to regular branching programs in which all of the transition functions $B_t$ are the same. They showed that if the graph is consistently labeled (equivalently, if we have a permutation branching program), then a seed length of $s=O(\log (nwd/\varepsilon ))$ suffices for the random walk to get within distance $\varepsilon$ of the uniform distribution on vertices, provided that the length *n* of the pseudorandom walk is polynomially larger than the mixing time of a truly random walk. (This “pseudo-mixing” property is nonstandard but has applications, including giving a simpler proof of Reingold’s Theorem that Undirected Connectivity is in deterministic logspace [12] and the construction of almost *k*-wise independent permutations [13].)

Next, Braverman et al. [14] analyzed the INW generator for regular branching programs of alphabet size $d=2$, and achieved seed length $s=O(\log n\cdot \log \log n + \log n \cdot \log (w/\varepsilon ))$, thereby improving the dependence on the length *n* from $O(\log ^2 n)$ to ${\tilde{O}}(\log n)$ for the standard pseudorandomness property. For the case of *permutation* branching programs of *constant width* $w=O(1)$ and alphabet size $d=2$, Koucký et al. [15] further improved the seed length to $s=O_{w}(\log n\cdot \log (1/\varepsilon ))$. The hidden constant in the $O_{w}(.)$ depended exponentially on the width *w*, but was subsequently improved to a polynomial by De [16] and Steinke [17].

Recently, Hoza et al. [18] turned their attention to permutation branching programs of *unbounded width*, and showed that the INW generator fools such programs in “spectral norm” with seed length $s=O\left( \log n\cdot \log \log n+\log n\cdot \log \left( 1/\varepsilon \right) +\log d\right)$. Here, fooling in spectral norm means that the $w\times w$ matrix of probabilities of going from each initial state to each final state under the generator has distance at most $\varepsilon$ in spectral norm from the same matrix under truly random inputs. $\varepsilon$-fooling in spectral norm can be shown to imply the standard notion of pseudorandomness for programs with a single accept state. Surprisingly, the seed length of Ref. [18] even beats the probabilistic method; indeed they show that a random function requires seed length $\Omega (n)$ to fool permutation branching programs of unbounded width and a single accept vertex with high probability. Subsequent to initial publication of this paper, Bogdanov et al. [19] achieved the same seed length for regular programs with a single accept vertex, at the cost of only obtaining a hitting set generator.

**Table 1:** Spectral analyses of the INW generator

Model	Seed length	Pseudorandomness	Reference
General	$O\left( \log n\cdot \log \left( nwd/\varepsilon \right) \right)$	Standard	[7]
Perm., same trans	$O\left( \log \left( nwd/\varepsilon \right) \right)$	Pseudo-mixing	[11]
Regular, $d=2$	$O\left( \log n\cdot \log \log n\,+\,\right.$ $\left. \log n\cdot \log \left( w/\varepsilon \right) \right)$	Standard	[14]
Regular	$O(\log n\cdot \log \log n \,+$ $\,\log n\cdot \log (1/\varepsilon )+\log d)$	Hitting	[19]
Permutation, $d=2$	$O_{w}(\log n\cdot \log (1/\varepsilon ))$	Standard	[15–17]
Permutation	$O(\log n\cdot \log \log n \,+$ $\,\log n\cdot \log (1/\varepsilon )+\log d)$	Spectral	[18]

We summarize the aforementioned analyses of the INW generator in Table 1. Let us elaborate on how all of these results are instantations of the INW generator. Specifically, the INW generator can be viewed as a template for a recursive construction of a PRG, where a PRG ${{\,\textrm{INW}\,}}_{i-1}$ generating $n_{i-1}=2^{i-1}$ output symbols is used to construct a PRG ${{\,\textrm{INW}\,}}_i$ generating $n_i=2^{i}$ output symbols, by running ${{\,\textrm{INW}\,}}_{i-1}$ twice on a pair of correlated seeds. The pair of seeds are chosen according to a random edge in an auxiliary expander graph $H_i$:

1 $$\begin{aligned} {{\,\textrm{INW}\,}}_i(e)={{\,\textrm{INW}\,}}_{i-1}(x) \cdot {{\,\textrm{INW}\,}}_{i-1}(y) \text { for each edge }e=(x,y)\,\text {of}\,H_i, \end{aligned}$$

where $\cdot$ denotes concatenation. Thus different choices of the sequence of graphs $H_1,H_2,\ldots ,H_{\log n}$ yield different instantiations of the INW generator. In all of the aforementioned works,^1^ the pseudorandomness property of the generator is proven using only the spectral expansion properties of the graphs $H_i$, namely requiring that all of the nontrivial normalized eigenvalues of $H_i$ have absolute value at most some value $\lambda _i$ for $i=1,\ldots ,\log n$. We call such an analysis a *spectral analysis* of the INW generator. Given a spectral analysis of the INW generator, the degrees of the expanders $H_i$ are then determined by the optimal relationship between expansion and degree $d_i=\text {poly}(1/\lambda _i)$ (see Proposition 2.6), which in turn determines the seed length of the final generator, namely

2 $$\begin{aligned} s=\Theta \left( \log \left( d\cdot d_1\cdot d_2\cdots d_{\log n}\right) \right) = \Omega \left( \log d+\sum _{i=1}^{\log n} \log (1/\lambda _i)\right) . \end{aligned}$$

### Our Results

Given the improved analyses of the INW generator described in Table 1, it is natural to wonder how much further these analyses can be pushed. In particular, can the INW generator $\varepsilon$-fool permutation branching programs of length *n*, width *w*, and alphabet size *d* with seed length matching the optimal seed length of $O(\log (nwd/\varepsilon ))$? Our main result is that the answer is no:

#### Theorem 1.5

(informally stated) Any spectral analysis of the INW generator for $\varepsilon$-fooling permutation branching programs of length *n*, width *w*, and alphabet size *d* requires seed length

$$\begin{aligned} s=\Omega \left( \log n\cdot \log \log (\min \{n,d\})+\log n\cdot \log (w/\varepsilon )+\log d\right) . \end{aligned}$$

Notice that this lower bound nearly matches the upper bounds in Table 1. In particular, we match the upper bound of Ref. [14] for regular branching programs, except that we get a $\log n\cdot \log \log n$ term only when $d = 2^{(\log n)^{\Omega (1)}}$ while they have such a term even when $d=2$. We also match the upper bounds of Ref. [15–17] for permutation branching programs of alphabet size $d=2$ and constant width $w=O(1)$.

For fooling with respect to spectral norm, we can get a lower bound of $\log n\cdot \log \log n$ whenever $w=n^{\Omega (1)}$, in particular matching the result of Ref. [18] for unbounded-width permutation branching programs:

#### Theorem 1.6

(informally stated) For $\varepsilon$-fooling in spectral norm, any spectral analysis of the INW generator for permutation branching programs of length *n*, width *w*, and alphabet size $d=2$ requires seed length

$$\begin{aligned} s=\Omega \left( \log n\cdot \log \log (\min \{n,w\})+\log n\cdot \log (1/\varepsilon )\right) . \end{aligned}$$

While our theorems are quite close to the upper bounds, they leave a few regimes where a spectral analysis of the INW generator could potentially yield an improved seed length. In particular, a couple of open questions stand out regarding the $\log n\cdot \log \log n$ terms in the bounds:

- Can we achieve seed length $O(\log n\cdot \log (w/\varepsilon ))$ for permutation (or even regular) branching programs of alphabet size $d=2$? When the alphabet size is $d=2$, the $\log \log (\min \{n,d\})$ term disappears in Theorem 1.5. However, the upper bound of Ref. [14] for regular branching programs still has an $O(\log n\cdot \log \log n)$ term, and the upper bounds of Ref. [15–17] only achieve a polynomial dependence on the width *w*.
- Can we achieve seed length $O(\log n\cdot \log (1/\varepsilon ))$ for permutation branching programs with a single accept vertex, alphabet size $d=2$, and width $w=n$ (or even unbounded width)? The best upper bound for this model is Ref. [18], which has an additional $O(\log n\cdot \log \log n)$ term. This term is necessary for fooling in spectral-norm by Theorem 1.6 but may not be necessary for the easier task of fooling programs with a single accept vertex.

A second opportunity for improvement is to go beyond spectral analysis of the INW generator, and exploit graphs $H_i$ with additional properties. To indicate that there is some hope for this, we include an observation showing that there *exists* an instantiation of the INW generator that achieves optimal seed length, even against more general ordered branching programs:

#### Theorem 1.7

For all $n,w,d \in \mathbb {N}$ and $\varepsilon >0$, there exists a sequence of graphs ${\mathcal {H}}$ such that the INW generator constructed with this sequence $\varepsilon$-fools ordered branching programs of length *n*, width *w* and alphabet size *d* and has seed length $O(\log (nwd/\varepsilon ))$.

This is an application of the Probabilistic Method, and so does not give an explicit PRG.

Our lower bounds also say nothing about constructions that deviate from the template of the INW generator, and better seed lengths can potentially be obtained by modifying the INW generator or using it as a tool in more involved constructions. Examples include the pseudorandom generator for width 3 ordered branching programs [10], which combines the INW generator with pseudorandom restrictions, and [22–26], which construct “weighted pseudorandom generators” with a better dependence on the error by taking linear combinations of the INW generator (or blends of the Nisan and INW generator).

### Techniques

Theorem 1.5 is really three separate lower bounds, which we state as separate theorems here to discuss the proof ideas separately. (The lower bound of $s=\Omega (\log d)$ is very simple.)

#### Theorem 1.8

(informally stated) Any spectral analysis of the INW generator for $(1-1/w^{\Omega (1)})$-fooling permutation branching programs of length *n*, width *w*, and alphabet size *d* requires seed length $s=\Omega (\log n\cdot \log w).$

Note that the lower bound holds for a very large error parameter, namely $\varepsilon =1-1/w^{\Omega (1)}$. In fact, it holds even for obtaining a *hitting-set generator*, where Definition 1.2 is relaxed to only require that $\mathop {\mathrm {\mathbb {E}}}\limits _{x \leftarrow U_{[d]^n}}[f(x)]>\varepsilon$ implies that $\mathop {\mathrm {\mathbb {E}}}\limits _{x \leftarrow U_{[S]}}[f({{\,\textrm{GEN}\,}}(x))]>0$.

To prove this Theorem 1.8, we show that most of the $\lambda _i$’s parameterizing the INW generator must have $\lambda _i < 1/w^{\Omega (1)}$, which implies the seed-length lower bound by Eq. (2). If that is not the case for some value of *i*, we construct an auxiliary graph $H_i$ to use in the INW generator (with $\lambda (H_i)\le \lambda _i$) such that a permutation branching program only needs width $\text {poly}(1/\lambda _i) \le w$ in order to perfectly distinguish a random edge in $H_i$ from a pair of vertices in $H_i$ that are not adjacent. Specifically, we can take $H_i$ to be an expander with degree $c_i=\text {poly}(1/\lambda _i)$ and $c_i^2$ vertices. To be able to use such a graph in most levels in the INW generator, we need to pad the number of vertices. We do this by taking a tensor product with a complete graph, which retains both the expansion of $H_i$ and the ability of a width *w* permutation branching program to distinguish edges and non-edges. We use complete graphs (with an appropriate edge labeling) for the remaining graphs $H_j$ in the INW generator, and argue a permutation branching program of width *w* can still distinguish the output from uniform.

#### Theorem 1.9

(informally stated) Any spectral analysis of the INW generator for $\varepsilon$-fooling permutation branching programs of length *n*, width $w=2$, and alphabet size *d* requires seed length $s=\Omega (\log n\cdot \log (1/\varepsilon )).$

To prove Theorem 1.9 we use a construction from Ref. [11] used to show the tightness of their analysis of the “derandomized square” operation on graphs. (Composing the INW generator with a permutation branching programs amounts to performing $\log n$ iterated derandomized square operations on the graph of the branching program.) Specifically, in order to show that each $\lambda _i$ satisfies $\lambda _i=O(\varepsilon )$, we consider a graph $H_i$ that has a self-loop probability of $\lambda _i$ but has $\lambda (H_i)\le \lambda _i$. When the self-loop is taken, it means that two consecutive subsequences of the output of the INW generator of length $2^{i-1}$ are equal to each other, by Eq. (1). Thus the permutation branching program of width 2 that computes the parity of the input bits on the union of those two subsequences will distinguish the output of the INW generator from uniform with advantage $\Omega (\lambda _i)$.

#### Theorem 1.10

(informally stated) Any spectral analysis of the INW generator for (1/20)-fooling permutation branching programs of length *n*, width $w=2$, and alphabet size *d* requires seed length $s=\Omega (\log n\cdot \log \log (\min \{n,d\})).$

To prove Theorem 1.10, we want to show that most of the $\lambda _i$’s must satisfy $\lambda _i \le O(1/\log n)$. For the overview, we assume that $d=n$. It suffices to prove that $\sum _{i=1}^{\log n} \lambda _i \le O(1)$. To do this, we again consider graphs $H_i$ that have a self-loop probability of $\lambda _i$, but rather than considering only one such graph, we use all of them in the INW generator. Intuitively, we want to show that the errors of $\Omega (\lambda _i)$ accumulate to lead to an overall error of $\Omega (\sum _i \lambda _i) > \varepsilon$. We consider a permutation branching program that corresponds to a random walk on a graph *G* with $w=2$ vertices that has a self-loop probability of approximately $1-1/n$. A truly random walk of length *n* on *G* will end at its start vertex with probability at most $1-n\cdot (1/n)\cdot (1-1/n)^{n-1} <.64$. We show that a pseudorandom walk using the INW generator with the graphs $H_i$ will end at its start vertex with probability at least .75. Specifically, we choose our edge and vertex labelings carefully so that the self-loops in the graphs $H_i$ cause random walks to backtrack with a high constant probability, so that it is as if we are typically doing random walks on *G* of length at most *n*/4.

Turning to Theorem 1.6, the only part of the lower bound that does not follow from the same arguments as above is the following:

#### Theorem 1.11

(informally stated) For 1/3-fooling in spectral norm, any spectral analysis of the INW generator for permutation branching programs of length *n*, width *w*, and alphabet size $d=2$ requires seed length $s=\Omega (\log n\cdot \log \log (\min \{n,w\})).$

The proof of Theorem 1.11 is similar to that of Theorem 1.10, but instead of considering random walks on a 2-vertex graph *G* with large degree *d*, we use a graph *G* of degree 2 and a large number of vertices. Specifically we take *G* to be the undirected cycle on $w=\Theta (\sqrt{n})$ vertices. The key point is that the truly random walk on the cycle mixes in $n=\Theta (w^2)$ steps in spectral norm. So a truly random walk of length *n* will differ from complete mixing by at most, say 1/3, in spectral norm, but due to backtracking, the pseudorandom walks using the INW generator will differ from complete mixing by at least 2/3 in spectral norm.

### Organization

In Sect. 2, we introduce formal definitions and give our general recipe for proving lower bounds. In Sect. 3, we prove Theorem 1.9, our lower bound in terms of the error of the pseudorandom generator. In Sect. 4, we show how the error incurred in different levels of the INW generator can accumulate, leading to Theorems 1.10 and 1.11. In Sect. 5, we prove Theorem 1.8, our lower bound in terms of the width. In Appendix A, we observe that this lower-bound technique gives stronger results for fooling general (e.g. non-regular) ordered branching programs, and in particular recovers the analysis of Brody and Verbin for bounds against width-3 ordered branching programs. In Appendix B, we prove Theorem 1.7, establishing the existence of graphs enabling the INW generator to achieve optimal seed length.

## Structure of Lower Bounds

We now give the general approach to proving our lower bounds. To define spectral analysis, we introduce notation related to labeled graphs and distributions.

### Definition 2.1

(One-way labeling [11]) A *one-way labeling* of a *d*-regular directed (multi)graph *G* assigns a label in [*d*] to each edge (*u*, *v*) such that for every vertex *u*, the labels of the outgoing edges of *u* are distinct. For *G* with a one-way labeling, let *G*[*u*, *i*] denote the vertex *v* such that (*u*, *v*) is labeled *i*. Furthermore, for ${\bar{y}}=(y_1,\dots ,y_k)\in [d]^k$ let $G^k[x,{\bar{y}}]$ be the vertex obtained from following the sequence of edge labels ${\bar{y}}$, i.e. $G^k[x,{\bar{y}}]=G[G[\ldots G[x,y_1],\ldots ,y_{k-1}],y_k]$.

For the remainder of the paper all graphs have one-way labelings.

- For all $w \in \mathbb {N}$, let $J_w$ be the *w*-regular graph on *w* vertices with the one-way labeling $J_w[x,y]=y$ for all $x,y\in [w]$ (i.e. the complete directed graph with self loops).
- For all $w \in \mathbb {N}$, let $I_w$ be the 1-regular graph with one-way labeling $I_w[x,0]=x$ for all $x\in [w]$, i.e. a single (directed) self loop on every vertex.

We occasionally write $J_*$ (resp. $I_*$) where the size of the graph is obvious from context. All logs are base-2, and we use the nonstandard definition that $[T]=\{0,\dots ,T-1\}$ for all $T\in \mathbb {N}$. In addition, we work with the random walk matrices of graphs, and the distribution induced by taking walks on graphs according to the output of a PRG.

### Definition 2.2

For a *d*-regular labeled graph *G* on *w* vertices and a label $y \in [d]$, let ${\textbf{W}}_G[y]\in \{0,1\}^{w\times w}$ be the matrix where entry (*u*, *v*) is 1 if and only if $G[u,y]=v$. Furthermore, we can define the *random walk matrix of G* as $\overline{{\textbf{W}}}_G=\mathop {\mathrm {\mathbb {E}}}\limits [{\textbf{W}}_G[U_{[d]}]]$. Furthermore, for a function ${{\,\textrm{GEN}\,}}:[S]\rightarrow [d]^k$, define

$$\begin{aligned}\overline{{\textbf{W}}_{G^k}\circ {{\,\textrm{GEN}\,}}} = \mathop {\mathrm {\mathbb {E}}}\limits [{\textbf{W}}_{G^k}[{{\,\textrm{GEN}\,}}(U_{[S]})]].\end{aligned}$$

Note that with this notation, $\overline{{\textbf{W}}}_{G^k}=(\overline{{\textbf{W}}}_G)^k$ for every *k*.

### Definition 2.3

For a *d*-regular digraph *G* on *w* vertices, define the *spectral expansion* of *G* as $\lambda (G)=\max _{x:x\perp 1,x\ne \vec {0}}\Vert x\overline{{\textbf{W}}}_G\Vert _2/\Vert x\Vert _2$.

We now formally define the INW PRG.

### Definition 2.4

Given $d_0\in \mathbb {N}$ and a set of graphs ${\mathcal {H}}=(H_1,\ldots ,H_\ell )$ where $\deg (H_i)=d_i$ and $|H_i|=\prod _{j=0}^{i-1}d_j$, the *INW generator constructed with* ${\mathcal {H}}$, denoted ${{\,\textrm{INW}\,}}_{\mathcal {H}}$ or ${{\,\textrm{INW}\,}}_\ell$ when the family is clear, is the function ${{\,\textrm{INW}\,}}_{\mathcal {H}}:[d_0]\times \dots \times [d_\ell ]\rightarrow [d_0]^{2^\ell }$ defined recursively where for $x\in [d_0]$ we have ${{\,\textrm{INW}\,}}_0(x)=x$ and for $(x,y)\in ([d_0]\times \dots \times [d_i], [d_{i+1}])$ we have

$$\begin{aligned} {{\,\textrm{INW}\,}}_{i+1}(x,y)=({{\,\textrm{INW}\,}}_i(x),{{\,\textrm{INW}\,}}_{i}(H_{i+1}[x,y])). \end{aligned}$$

The seed length of ${{\,\textrm{INW}\,}}_{\mathcal {H}}$ is thus $\log \left( \prod _{i=0}^\ell d_i\right)$.^2^

We then define an analysis of the INW PRG that only “knows about” the spectral gap of the auxiliary graphs. For the remainder of the paper (with the exception of Appendix B) we assume all auxiliary graphs *H* are undirected,^3^ so we can assume ${\textbf{W}}_H$ has a basis of eigenvectors.

### Definition 2.5

For $d_0\in \mathbb {N}$ and $\lambda _1,\ldots ,\lambda _\ell \ge 0$, let ${{\,\textrm{INW}\,}}(d_0,\lambda _1,\ldots ,\lambda _\ell )$ be the set of INW PRGs ${{\,\textrm{GEN}\,}}:[S]\rightarrow [d_0]^{2^\ell }$ constructed with auxiliary undirected regular graphs $H_1,\ldots ,H_\ell$ where $\lambda (H_i)\le \lambda _i$ for all *i*. We say ${{\,\textrm{INW}\,}}(d_0,\lambda _1,\ldots ,\lambda _\ell )$ $\varepsilon$-fools a class of functions ${\mathcal {F}}$ if every ${{\,\textrm{GEN}\,}}\in {{\,\textrm{INW}\,}}(d_0,\lambda _1,\ldots ,\lambda _\ell )$ $\varepsilon$-fools every $f\in {\mathcal {F}}$. Furthermore, define $s_{{{\,\textrm{INW}\,}}}(d_0,\lambda _1,\ldots ,\lambda _\ell )$ as the minimal seed length of all PRGs in ${{\,\textrm{INW}\,}}(d_0,\lambda _1,\ldots ,\lambda _\ell )$. We call the set $(\lambda _1,\ldots ,\lambda _\ell )$ a *constraint*, and say a family of graphs $(H_1,\ldots ,H_\ell )$ *satisfies* the constraint if $\lambda (H_i)\le \lambda _i$ for all *i*.

Given a family of INW PRGs, we can derive a lower bound on the seed length via the relation between degree and maximum expansion, as given by the following standard fact.

### Proposition 2.6

(see e.g. [27]) Let *G* be an undirected *d*-regular graph on *V* vertices. Then

$$\begin{aligned} \lambda (G)\ge \frac{1}{\sqrt{d}}\sqrt{\frac{V-d}{V-1}}. \end{aligned}$$

In particular $$d\ge \min \{2/\lambda (G)^2,(V+1)/2\}$$.

That is, the degree must be at least polynomially related to $1/\lambda (G)$ [as assumed in the seed-length calculation in Eq. (2)], unless *d* is very close to the number of vertices. To deal with the latter case in our seed-length lower bounds, we will remove the terms corresponding to $\lambda _i$’s where the $2/\lambda (G)^2>(V+1)/2$, yielding the following:

### Lemma 2.7

Given ${{\,\textrm{INW}\,}}(d_0,\lambda _1,\ldots ,\lambda _\ell )$, there is a set $S\subseteq \{1,\ldots ,\ell \}$ with $|S|\le 2\log (\ell )+2\log \log (1/\lambda _{\min })$ where $\lambda _{\min }=\min \{\lambda _1,\ldots ,\lambda _\ell \}$ such that

$$\begin{aligned}s_{{{\,\textrm{INW}\,}}}(d_0,\lambda _1,\ldots ,\lambda _\ell )=\Omega \left( \sum _{i \in \{1,\ldots ,\ell \}\setminus S}\log (1/\lambda _i)\right) .\end{aligned}$$

### Proof

Let $t=\log (1/\lambda _{\min })$. Recall that $s_{{{\,\textrm{INW}\,}}}(d_0,\lambda _1,\ldots ,\lambda _\ell )= \log (d_0\cdot m_p)$, where $m_p$ is the minimum product of degrees over all sets of auxiliary graphs $H_1,\ldots ,H_\ell$ with the required spectral expansion. Let $H_1,\ldots ,H_\ell$ be such a minimal family, let $d_i = \deg (H_i)$ for each *i*, and let *R* be the set of “dense” graphs, i.e., the set of $i \in \{1, \dots , \ell \}$ such that $2/\lambda (H_i)^2 > (|H_i| + 1) / 2$. We now break into two cases based on |*R*|:

1. First, suppose $|R| \ge 2 \log (\ell t)$. Then set $S = \emptyset$. To prove that this works, let $p_i = \prod _{j = 0}^i d_j$, so $|H_i| = p_{i - 1}$. Observe that if $i \in R$, then $d_i \ge (|H_i| + 1)/2 = (p_{i - 1} + 1)/2$ by Proposition 2.6. Therefore,
  $$\begin{aligned} p_i = p_{i - 1} \cdot d_i \ge \frac{1}{2} \cdot p_{i - 1} \cdot (p_{i - 1} + 1) \ge p_{i - 1}^{3/2}, \end{aligned}$$
  where the last step uses the fact that $p_{i - 1} \ge d_0 \ge 2$. Consequently, for each $i \in R$, we have $\log p_i \ge \frac{3}{2} \cdot \log p_{i - 1}$, and so $\log p_{\ell } \ge (3/2)^{|R|} \ge (3/2)^{2 \log (\ell t)} = \Omega (\ell \cdot t)$.
2. Otherwise, let $S = R$. For every $i \notin S$, we have $\deg (H_i) \ge 2/\lambda (H_i)^2 \ge 2/\lambda _i^2$ by Proposition 2.6. Thus,
  $$\begin{aligned} m_p \ge \prod _{i \in S^c} \deg (H_i) \ge \prod _{i \in S^c} \frac{1}{\lambda _i^2}. \end{aligned}$$
  Thus we bound $s_{{{\,\textrm{INW}\,}}} = \log (d_0 \cdot m_p)$ as desired.

$$\square $$

We remark that, fixing *d*, $s_{{{\,\textrm{INW}\,}}}(d,\cdot )$ is monotonic with respect to every parameter, since a set of graphs that satisfies a constraint $(\lambda _1,\ldots ,\lambda _\ell )$ also satisfies every looser set of constraints.

Finally, we define the tensor product of graphs, and recall a basic fact about their expansion, as we will construct some auxiliary graphs via tensoring a small expander with the complete graph.

### Definition 2.8

Given a pair of labeled graphs *G*, *H* on $w_1,w_2$ vertices with degrees $d_1,d_2$ respectively, define the *tensor product* $G\otimes H$ to be the $d_1d_2$-regular graph on $w_1w_2$ vertices with neighbor relation $(G\otimes H)[(u,v),(e_1,e_2)]=(G[u,e_1],H[v,e_2])$.

### Proposition 2.9

(see e.g. [28, Lemma 4.33]) Let *G*, *H* be undirected regular graphs. Then $\lambda (G\otimes H)=\max (\lambda (G),\lambda (H))$.

## Dependence on Error

In this section, we prove Theorem 1.9, establishing a lower bound on the seed length as a function of the error of the generator.

### Theorem 3.1

(Formal Statement of Theorem 1.9) For every *d* and $n=2^\ell$ and $\varepsilon \ge 2^{-n/2}$ and $\lambda _1,\ldots ,\lambda _\ell \ge 0$, if ${{\,\textrm{INW}\,}}(d,\lambda _1,\ldots ,\lambda _\ell )$ $\varepsilon$-fools ordered permutation branching programs of length *n*, width 2, and alphabet size *d*, then $s_{{{\,\textrm{INW}\,}}}(d,\lambda _1,\ldots ,\lambda _\ell )=\Omega (\log (1/\varepsilon ) \cdot (\log (n)-\log \log (1/\varepsilon )))$.

This follows as a consequence of the following lemma, which essentially states that constructing an $\varepsilon$-biased space using the spectral INW generator requires constraining all spectral gaps to be $O(1/\varepsilon )$.

### Lemma 3.2

For all $d\in \mathbb {N}$ and $\varepsilon >0$, for every constraint $(\lambda _1,\ldots ,\lambda _\ell )$ where there is *r* such that $\lambda _r>3\varepsilon$, there is a family of auxiliary graphs ${\mathcal {H}}=(H_1,\ldots ,H_\ell )$ where $\lambda (H_i) \le \lambda _i$ and an alphabet size *d*, width 2, length $n=2^\ell$ permutation branching program *B* such that ${{\,\textrm{INW}\,}}_{\mathcal {H}}$ fails to $\varepsilon$-fool *B*.

To prove the lemma, we define convex combinations of graphs on the same vertex set.

### Definition 3.3

For $G,G'$ arbitrary *d*-regular graphs on *n* vertices, and $\lambda = a/b \in \mathbb {Q}\cap [0,1]$, let $H=\lambda G+(1-\lambda )G'$ be the $(d\cdot b)$-regular directed graph on *n* vertices where for $x\in [n]$ and $(y,c)\in [d]\times [b]$:

$$\begin{aligned}H[x,(y,c)] = {\left\{ \begin{array}{ll} G[x,y] &  c< a\\ G^{\prime }[x,y] &  c\ge a \end{array}\right. }\end{aligned}$$

We remark that with this definition, $\overline{{\textbf{W}}}_{H} = \lambda \overline{{\textbf{W}}}_G+(1-\lambda )\overline{{\textbf{W}}}_{G^{\prime }}$. We implicitly extend this to convex combinations of graphs with non-equal degrees $d,d^{\prime }$ by duplicating edges so both graphs have degree $\operatorname {LCM}(d,d^{\prime })$.

We can then construct a bad family of graphs and a distinguisher.

### Proof of Lemma 3.2

Let $\mu$ be a rational number in $(3\varepsilon ,\lambda _r]$ and let $K=2^{2^{r-1}}$ and define

$$\begin{aligned}H=\mu I_K+(1-\mu )J_K.\end{aligned}$$

Then define the family ${\mathcal {H}}=(J_2, J_4,\ldots ,J_{2^{2^{r-2}}},H,J_*,\ldots ,J_*)$. It is clear ${\mathcal {H}}$ satisfies the constraint.

Now let *B* be the length *n*, width 2, alphabet size *d* permutation branching program where, letting $T=\{0,\ldots ,\lceil d/2\rceil \}$, we have

$$\begin{aligned}B(\sigma )=\bigoplus _{i=1}^{2^r}\mathbb {I}[\sigma _i \in T].\end{aligned}$$

Let $\delta := \Pr [B(U_{{[d]^n}})=1]$. Furthermore, for every seed $\sigma =(x,u,*)$ we have

$$\begin{aligned}{{\,\textrm{INW}\,}}_{\mathcal {H}}(\sigma )_{1..2^r}={{\,\textrm{INW}\,}}_r((x,u))=({{\,\textrm{INW}\,}}_{r-1}(x),{{\,\textrm{INW}\,}}_{r-1}(H[x,u])) = (x,H[x,u]).\end{aligned}$$

From our definition of *H*, with probability $1-\mu$ over the random seed $\sigma$ the first $2^r$ bits output are (*x*, *y*) where (*x*, *y*) is distributed uniformly over $\{0,1\}^{2^r}$, and with probability $\mu$ the first $2^r$ bits of output are (*x*, *x*), which has parity zero for all *x*. Therefore, letting [*S*] be the seed space of ${{\,\textrm{INW}\,}}_{\mathcal {H}}$,

$$\begin{aligned} \Pr [B({{\,\textrm{INW}\,}}_{\mathcal {H}}(U_{[S]}))=1]= \delta \cdot (1-\mu )+0\cdot \mu =\delta - \mu \cdot \delta < \delta - \varepsilon \end{aligned}$$

where the final step uses that $\delta \in (1/3,2/3)$, and so ${{\,\textrm{INW}\,}}_{\mathcal {H}}$ fails to $\varepsilon$-fool *B*. $\square$

We can then prove Theorem 3.1.

### Proof of Theorem 3.1

Applying Lemma 3.2, every family ${{\,\textrm{INW}\,}}(d,\lambda _1,\ldots ,\lambda _\ell )$ that $\varepsilon$-fools length *n*, width 2, alphabet size *d* permutation branching programs has $\lambda _i\le 3\varepsilon$ for all *i*, so we obtain $s_{{{\,\textrm{INW}\,}}}(d,\lambda _1,\ldots ,\lambda _\ell )\ge s_{{{\,\textrm{INW}\,}}}(d,3\varepsilon ,\ldots ,3\varepsilon )$. Now fix a family of graphs ${\mathcal {H}}=(H_1,\ldots ,H_\ell )$ satisfying this constraint, and let $d_i = \deg (H_i)$ and $p_i = \prod _{j\le i}d_i$. Let $t=\lfloor \log \log (1/\varepsilon )\rfloor$ and note $t\le \ell$ by assumption on $\varepsilon$. We show that $\log (p_t) \ge \Omega (\log (1/\varepsilon ))$ by considering two cases:

- If there is some $i \in [t]$ such that $d_i \ge 2/\varepsilon ^2$, then clearly $\log (p_t) \ge \log (d_i) \ge \Omega (\log (1/\varepsilon ))$.
- Otherwise, by Proposition 2.6, we have $d_i \ge (p_{i - 1} + 1) / 2$ for every $i \in \{1, \dots , t - 1\}$. Therefore,
  $$\begin{aligned} p_i = p_{i - 1} \cdot d_i \ge \frac{1}{2} \cdot p_{i - 1} \cdot (p_{i - 1} + 1). \end{aligned}$$
  Since $p_0 = d_0 \ge 2$, the inequality above implies $p_1 \ge 3$ and $p_2 \ge 6$, hence $\log (p_2) > 2 = 2^0 + 1$. In subsequent steps, the inequality above implies $p_i \ge \frac{1}{2} p_{i - 1}^2$ and hence $\log (p_i) \ge 2 \log (p_{i - 1}) - 1$. Consequently, $\log (p_t) \ge 2^{t - 2} + 1 = \Omega (\log (1/\varepsilon ))$.

Thus, for every $i > t$, every level contributes at least $\Omega (\log (1/\varepsilon ))$ bits of seed, so we obtain the lower bound of $\Omega (\log (1/\varepsilon ) \cdot (\log (n) - \log \log (1/\varepsilon )))$. $\square$

## Accumulation of Error

In this section, we prove the lower bounds on seed length $\Omega (\log n\cdot \log \log \min \{n,d\})$ and $\Omega (\log n\cdot \log \log \min \{n,w\})$ from Theorems 1.5 and 1.6, respectively. As discussed in the introduction, in both of these lower bounds, we wish to show that that the error $\Omega (\lambda _i)$ demonstrated in Sect. 3 actually accumulates to give an error of $\varepsilon =\Omega (\sum _i \lambda _i)$, which will imply that most of the $\lambda _i$’s are $O(1/\log n)$ and hence we require seed length $\Omega (\log n\cdot \log \log n)$. For the standard notion of pseudorandomness, we will be able to argue this when the alphabet size of the branching programs is polynomially related to *n*, and for fooling in spectral norm, we will be able to argue it when the width of the branching program is polynomially related to *n*.

### The INW PRG On Reversible Graphs

We will analyze the distribution of the output of the INW PRG over graphs, taking the transition function of the branching program to equal that of the graph. We recall the connection between consistently labeled graphs and permutation branching programs.

#### Definition 4.1

A *d*-regular labeled graph *G* on *w* vertices is *consistently labeled* if $G[v,i]=G[v^{\prime },i]$ implies $v=v^{\prime }$ for all $v,v^{\prime }\in [w]$, $i\in [d]$. Equivalently, each edge label $i\in [d]$ defines a permutation over [*w*].

#### Remark 4.2

Given a *d*-regular consistently labeled graph *G* on *w* vertices and $n\in \mathbb {N}$, the branching program $G^{(n)}$ of length *n*, width *w* and alphabet size *d* with transition functions $G_1(v,b)=\cdots =G_n(v,b)=G[v,b]$ is a permutation branching program.

To prove Theorems 1.5 and 1.6, we introduce a graph property that will be satisfied by the graphs we use as our distinguishing permutation branching programs. Furthermore, given such a graph we construct a family of expanders such that the INW PRG behaves as if it is taking walks that are a constant factor shorter than truly random, and are thus distinguishable.

#### Definition 4.3

A *d*-regular labeled graph *G* on *w* vertices is *reversible* if there exists an involution $\pi :[d]\rightarrow [d]$ such that for every edge label $\sigma \in [d]$ and vertex $v \in [w]$ we have $G^2[v,(\sigma ,\pi (\sigma ))]=G[G[v,\sigma ],\pi (\sigma )]=v$, i.e. ${\textbf{W}}_{G^2}[(\sigma ,\pi (\sigma ))]={\textbf{I}}$ for all $\sigma$.

We remark that this notion can be considered a directed analogue of Ta-Shma’s notion of a “locally invertible graph” [29].

Furthermore, given an involution $\pi$ and an edge sequence $\sigma =(\sigma _1,\ldots ,\sigma _m)$, define $\pi (\sigma ){\mathop {=}\limits ^{\text {def}}}(\pi (\sigma _m),\ldots ,\pi (\sigma _1))$. Then reversibility extends to arbitrary edge sequences.

#### Lemma 4.4

Given a *d*-regular reversible graph *G* with involution $\pi$, for every vertex *v* and edge sequence $\sigma \in [d]^m$, $G^{2m}[v,(\sigma ,\pi (\sigma ))]=v$.

#### Proof

This follows from induction on *m*. The case $m=1$ is clear from the definition, and assuming it holds for $m-1$, fix arbitrary *v* and $\sigma \in [d]^m$. We have

$$\begin{aligned} G^{2m}[v,(\sigma ,\pi (\sigma ))]&= G^{m-1}[G[G[G^{m-1}[v,\sigma _{1..m-1}],\sigma _m],\pi (\sigma _m)],\pi (\sigma _{1..m-1})]\\&= G^{m-1}[G^{m-1}[v,\sigma _{1..m-1}],\pi (\sigma _{1..m-1})] =v, \end{aligned}$$

so the inductive step holds. $\square$

Now given a reversible graph and a constraint set that is not too restrictive, we can construct an INW PRG that performs in a highly structured fashion. Intuitively, each generator output will consist of a mixture of truly random steps and steps that are “backtracked” and thus do not contribute to mixing. These backtracked steps will wipe out at least 3/4 of progress with high probability over the seed.

#### Lemma 4.5

Let *G* be a *d*-regular reversible graph and $(\lambda _i)_{\ell }$ a constraint where $\sum _{i=1}^\ell \lambda _i\ge 8$. Then there is a family of auxiliary graphs ${\mathcal {H}}=(H_1,\ldots ,H_\ell )$ where $\lambda (H_i)\le \lambda _i$ such that ${{\,\textrm{INW}\,}}_{\mathcal {H}}$ satisfies:

- $\overline{{\textbf{W}}_{G^{2^\ell }}\circ {{\,\textrm{INW}\,}}_{\mathcal {H}}}$ is a convex combination of $\overline{{\textbf{W}}}_G^0,\ldots ,\overline{{\textbf{W}}}_G^{2^\ell }$.
- In this convex combination, the sum of coefficients on $\overline{{\textbf{W}}}_G^0,\ldots ,\overline{{\textbf{W}}}_G^{2^\ell /4}$ is at least .99.

Our strategy is to use the fact that the graph is reversible to cause PRG outputs to backtrack with high probability. To do so, we define a property that each level of our PRG construction will satisfy.

#### Definition 4.6

Given an involution $\pi :[d]\rightarrow [d]$, a generator ${{\,\textrm{GEN}\,}}:[S]\rightarrow [d]^r$ is *balanced with respect to* $\pi$ if $\Pr [{{\,\textrm{GEN}\,}}(U_{[S]})=v] = \Pr [{{\,\textrm{GEN}\,}}(U_{[S]})=\pi (v)]$ for every $v \in [d]^r$.

We are now prepared to prove the lemma. We iteratively construct the PRG to comply with the constraint, while backtracking as many steps as possible.

#### Proof of Lemma 4.5

Assume without essential loss of generality that $\lambda _i$ is rational for every *i*. Let ${{\,\textrm{INW}\,}}_0:[d]\rightarrow [d]$ be the trivial PRG that outputs its input. At each step we maintain that ${{\,\textrm{INW}\,}}_i$ is balanced with respect to $\pi$, which is clearly satisfied for level 0.

Given ${{\,\textrm{INW}\,}}_i:[S_i]\rightarrow [d]^{2^i}$, we show how to construct ${{\,\textrm{INW}\,}}_{i+1}:[S_{i+1}]\rightarrow [d]^{2^{i+1}}$. For every output $t \in [d]^{2^i}$, let

$$\begin{aligned}\supseteq R_t={{\,\textrm{INW}\,}}_i^{-1}(t)\end{aligned}$$

be the seeds that cause the generator to output *t*. We have by assumption that $|R_t|=|R_{\pi (t)}|$ for all *t*. Let $M_t$ be an arbitrary bijection between $R_t$ and $R_{\pi (t)}$ and define *M* as the 1-regular graph on $[S_i]$ that maps $v\in R_t$ to $M_t(v)$ for every *v* and *t*. Then define

$$\begin{aligned}H_{i+1}=\lambda _{i+1} M+(1-\lambda _{i+1})J_*.\end{aligned}$$

And define ${{\,\textrm{INW}\,}}_{i+1}$ using this graph. Then the INW PRG constructed with this graph remains balanced.

#### Claim 4.7

${{\,\textrm{INW}\,}}_{i+1}(x,u)=\left( {{\,\textrm{INW}\,}}_{i}(x),{{\,\textrm{INW}\,}}_i\left( H_{i+1}[x,u]\right) \right)$ is balanced with respect to $\pi$.

#### Proof

Fix an arbitrary output $(a,b) \in [d]^{2^i}\times [d]^{2^i}$. We have

$$\begin{aligned}&\Pr \left[ {{\,\textrm{INW}\,}}_{i+1}\left( U_{\left[ S_{i + 1}\right] }\right) =(a,b)\right] \\&\quad = \lambda _{i+1}\Pr _{x\leftarrow U_{[S_i]}}\left[ {{\,\textrm{INW}\,}}_i(x)=a\wedge {{\,\textrm{INW}\,}}_i\left( M[x,0]\right) =b\right] \\&\quad + (1-\lambda _{i+1})\Pr _{x\leftarrow U_{[S_i]},y\leftarrow U_{[S_i]}}\left[ {{\,\textrm{INW}\,}}_i(x)=a\wedge {{\,\textrm{INW}\,}}_i(y)=b\right] \\&=: \lambda _{i+1}\cdot \alpha _{a,b}+(1-\lambda _{i+1})\cdot \beta _{a,b}. \end{aligned}$$

Thus, it suffices to show $\alpha _{a,b}=\alpha _{\pi (b),\pi (a)}$ and $\beta _{a,b}=\beta _{\pi (b),\pi (a)}$.

- If either $\alpha _{a,b}$ or $\alpha _{\pi (b),\pi (a)}$ is nonzero, we must have $b=\pi (a)$ from the way *M* was constructed. Thus, $(a,b)=(a,\pi (a))=(\pi (b),\pi (a))$ so $\alpha _{a,b}=\alpha _{\pi (b),\pi (a)}$.
- Let $\beta _a:=\Pr _{U_S}[{{\,\textrm{INW}\,}}_i(U_S)=a]$. By the inductive hypothesis $\beta _a=\beta _{\pi (a)}$ and $\beta _b=\beta _{\pi (b)}$. Thus, $\beta _{a,b}=\beta _a\beta _b=\beta _{\pi (b)}\beta _{\pi (a)}=\beta _{\pi (b),\pi (a)}$.

$$\square $$

Finally, for every $1 \le i \le \ell$, we have $\lambda (H_{i})\le \lambda _{i}\cdot \lambda (M)\le \lambda _i$ since the complete graph falls out, so the family satisfies the constraint. We then analyze the distribution of outputs of the PRG. Since ${{\,\textrm{INW}\,}}_0$ is the trivial PRG we have $\overline{{\textbf{W}}_G\circ {{\,\textrm{INW}\,}}_0}=\overline{{\textbf{W}}}_G$.

#### Claim 4.8

For all $$i\in [\ell ]$$,

$$\begin{aligned} \overline{{\textbf{W}}_{G^{2^{i+1}}}\circ {{\,\textrm{INW}\,}}_{i+1}}=\lambda _{i+1} {\textbf{I}}_{|G|}+(1-\lambda _{i+1})(\overline{{\textbf{W}}_{G^{2^{i}}}\circ {{\,\textrm{INW}\,}}_{i}})^2. \end{aligned}$$

#### Proof

Fixing an arbitrary vertex *v* in *G*, we compute the distribution of $G[v,{{\,\textrm{INW}\,}}_{i+1}(\sigma )]$ over a random seed $\sigma =(x,u)\leftarrow U_{[S_{i+1}]}$ of ${{\,\textrm{INW}\,}}_{i+1}$. From our construction of $H_{i+1}$, with probability $\lambda _{i+1}$ over the random seed this corresponds to a neighbor in the graph *M*, so we have

$$\begin{aligned}{{\,\textrm{INW}\,}}_{i+1}(\sigma )=({{\,\textrm{INW}\,}}_i(x),{{\,\textrm{INW}\,}}_i(H_{i+1}[x,u]))=(t,\pi (t))\end{aligned}$$

for some $t\in [d]^{2^i}$, and so $G[v,{{\,\textrm{INW}\,}}_{i+1}(\sigma )]=v$. Otherwise, with probability $1-\lambda _{i+1}$ over the random seed $H_{i+1}[x,u]$ corresponds to a neighbor in $J_*$, so we have

$$\begin{aligned}{{\,\textrm{INW}\,}}_{i+1}(\sigma )=({{\,\textrm{INW}\,}}_i(x),{{\,\textrm{INW}\,}}_i(H_{i+1}[x,u]))=({{\,\textrm{INW}\,}}_i(x),{{\,\textrm{INW}\,}}_i(y))\end{aligned}$$

with *x*, *y* independent and uniformly distributed over $U_{[S_i]}$, so the result follows. $\square$

Inductively define a sequence of integer-valued random variables $K_0, \dots , K_{\ell }$ as follows.

- $K_0 = 1$ with probability one.
- Let $0 < i \le \ell$. With probability $\lambda _i$, the variable $K_i$ is equal to 0, and with probability $1 - \lambda _i$, the variable $K_i$ is the sum of two independent copies of $K_{i - 1}$.

By induction, Claim 4.8 implies that $\overline{{\textbf{W}}_{G^{2^i}} \circ {{\,\textrm{INW}\,}}_i} = \mathop {\mathrm {\mathbb {E}}}\limits _{K_i}\left[ {\overline{{\textbf{W}}}}_G^{K_i}\right]$ for each *i*. This shows that $\overline{{\textbf{W}}_{G^{2^{\ell }}} \circ {{\,\textrm{INW}\,}}_{\ell }}$ is a convex combination over ${\overline{{\textbf{W}}}}^0_G, \dots , {\overline{{\textbf{W}}}}_G^{2^\ell }$, and to bound the coefficients, we must bound $\Pr [K_{\ell } \le 2^{\ell } / 4]$. Observe that $\mathop {\mathrm {\mathbb {E}}}\limits [K_0] = 1$ and $\mathop {\mathrm {\mathbb {E}}}\limits [K_i] = (1 - \lambda _i) \cdot 2 \cdot \mathop {\mathrm {\mathbb {E}}}\limits [K_{i - 1}]$ for $i > 0$. Therefore,

$$\begin{aligned} \mathop {\mathrm {\mathbb {E}}}\limits [K_{\ell }] = 2^{\ell } \cdot \prod _{i = 1}^{\ell } (1 - \lambda _i) \le 2^{\ell } \cdot \prod _{i = 1}^{\ell } e^{-\lambda _i} = 2^{\ell } \cdot e^{-\lambda _1 - \lambda _2 - \dots - \lambda _{\ell }} \le 2^{\ell } \cdot e^{-8}. \end{aligned}$$

Consequently, by Markov’s inequality, $\Pr [K_{\ell } > 2^{\ell } / 4] \le 4 \cdot e^{-8} < 0.01$. $\square$

### Branching Programs of Large Alphabet Size

We first give the formal statement of the theorem:

#### Theorem 4.9

(Formal Statement of Theorem 1.10) For every $n=2^\ell$ and $\lambda _1,\ldots ,\lambda _\ell \ge 0$, if ${{\,\textrm{INW}\,}}(d,\lambda _1,\ldots ,\lambda _\ell )$ (1/20)-fools ordered permutation branching programs of length *n*, width 2, and alphabet size *d*, then we have $s_{{{\,\textrm{INW}\,}}}(d,\lambda _1,\ldots ,\lambda _\ell )=\Omega (\log n\cdot \log \log (\min \{n,d\}))$.

We can now prove the key lemmas, the first being the constraint against polynomial alphabet size permutation branching programs of width 2.

#### Lemma 4.10

For every $n=2^\ell$ (for $\ell \ge 4$) and $d\ge n$ and every constraint $(\lambda _1,\ldots ,\lambda _\ell )$ where $\sum _{i=1}^\ell \lambda _i \ge 8$, there is a family of auxiliary graphs ${\mathcal {H}}=(H_1,\ldots ,H_\ell )$ where $\lambda (H_i) \le \lambda _i$ and a length *n*, width 2, alphabet size *d* permutation branching program *B* such that the INW generator constructed with ${\mathcal {H}}$ fails to (1/20)-fool *B*.

#### Proof

Let *G* be the *d*-regular graph on $\mathbb {Z}_2$ with the following neighbor relation:

$$\begin{aligned} G[v,b] = {\left\{ \begin{array}{ll} v+1 &  \text {if } b \equiv n-1 \pmod {n} \\ v &  \text {else.}\end{array}\right. } \end{aligned}$$

Let $\delta \in (\frac{1}{2n},\frac{1}{n}]$ be the probability of taking a non-self-loop step.

We will work with walks of length *n* over this graph, equivalent to computation on the length *n*, alphabet size *d* permutation branching program $B=G^{(n)}$ as in Remark 4.2.

It is easy to see that *G* is reversible (in fact with $\pi$ the identity function), so we apply Lemma 4.5 with *G* and $(\lambda _1,\ldots ,\lambda _\ell )$ and obtain a PRG ${{\,\textrm{INW}\,}}_{\mathcal {H}}$ where ${\mathcal {H}}$ satisfies the constraint.

To obtain the separation, we examine the probability that a random output of ${{\,\textrm{INW}\,}}_{\mathcal {H}}$ ends at state 0 from state 0 (i.e. $(\overline{{\textbf{W}}_{G^{(n)}}\circ {{\,\textrm{INW}\,}}_{\mathcal {H}}})_{0,0}$), compared to the equivalent probability over truly random input (i.e. $(\overline{{\textbf{W}}}_{G^{(n)}})_{0,0}=(\overline{{\textbf{W}}}_G^{(n)})_{0,0}$).

The probability a random walk of length *n* from state 0 in *G* ends at state 0 is upper bounded by 1 minus the probability that such a walk takes exactly one non-self loop step. Recall that $\text {Bin}(m,p,t)$ is the probability of obtaining *t* heads from *m* iid Bernoulli(*p*) draws. Therefore,

$$\begin{aligned} \left( \overline{{\textbf{W}}}_{G^{(n)}}\right) _{0,0} \le 1- \text {Bin}(n,\delta ,1) = 1- n\delta \left( 1-\delta \right) ^{n-1}. \end{aligned}$$

One can show that the derivative of the right-hand side with respect to $\delta$ (holding *n* fixed) is nonpositive for all $\delta \le \frac{1}{n}$. Since $\delta \in \left( \frac{1}{2n}, \frac{1}{n}\right]$, it follows that

$$\begin{aligned}&\left( \overline{{\textbf{W}}}_{G^{(n)}}\right) _{0,0} \le 1 - \frac{1}{2} \cdot \left( 1 - \frac{1}{2n}\right) ^{n - 1} \\&\quad = 1 - \frac{1}{2} \cdot \left( \left( 1 - \frac{1}{2n}\right) ^{(n - 1)/15}\right) ^{15} \\&\quad \le 1 - \frac{1}{2} \cdot \left( 1 - \frac{n - 1}{30 n}\right) ^{15}\quad \text {(Bernoulli's inequality)} \\&\quad< 1 - \frac{1}{2} \cdot \left( 1 - \frac{1}{30}\right) ^{15} < 0.7. \end{aligned}$$

(Note that we were able to apply Bernoulli’s inequality because we assumed $\ell \ge 4$ and hence $(n - 1) / 15 \ge 1$.) Intuitively, in the PRG output no backtracked section can possibly change the parity of the state, so $(\overline{{\textbf{W}}_{G^{(n)}}\circ {{\,\textrm{INW}\,}}_{\mathcal {H}}})_{0,0}$ is at least the probability that none of the non-backtracked steps (which are truly random) traverse edges in the cycle. Formally, for all $m\in \mathbb {N}$ we have $(\overline{{\textbf{W}}}_{G}^m)_{0,0}\ge \text {Bin}(m,\delta ,0)$. Since this bound is monotonically decreasing with *m*, we lower bound $(\overline{{\textbf{W}}_{G^{(n)}}\circ {{\,\textrm{INW}\,}}_{\mathcal {H}}})_{0,0}$ by Lemma 4.5:

$$\begin{aligned} \left( \overline{{\textbf{W}}_{G^{(n)}}\circ {{\,\textrm{INW}\,}}_{\mathcal {H}}}\right) _{0,0}&\ge \frac{1}{100}\text {Bin}\left( n,\delta ,0\right) +\frac{99}{100}\text {Bin}\left( n/4,\delta ,0\right) \\&\ge \frac{1}{100}\text {Bin}\left( n,1/n,0\right) +\frac{99}{100}\text {Bin}\left( n/4,1/n,0\right) \\&\ge \frac{99}{100}\left( 1-1/n\right) ^{n/4}\\&\ge .75 \end{aligned}$$

Therefore $\left( \overline{{\textbf{W}}_{G^{(n)}}\circ {{\,\textrm{INW}\,}}_{\mathcal {H}}}\right) _{0,0}-\left( \overline{{\textbf{W}}}_{G^{(n)}}\right) _{0,0} >.05$ and we have an $\Omega (1)$ separation as desired. $\square$

We can then use this lemma to prove the main result.

#### Proof of Theorem 4.9

Let $t=\lfloor \log (\min \{n,d\})\rfloor$ and fix some constraint $\left( \lambda _1,\ldots ,\lambda _\ell \right)$ such that ${{\,\textrm{INW}\,}}(d,\lambda _1,\ldots ,\lambda _\ell )$ (1/20)-fools the model.

#### Claim 4.11

Every block $\left( \lambda _i,\ldots ,\lambda _{i+t-1}\right)$ satisfies $\sum _{j=i}^{i+t-1}\lambda _j<8$.

#### Proof

Note that given $(\lambda _i,\ldots ,\lambda _{i+t-1})$ with $\sum _{j=i}^{i+t-1}\lambda _i\ge 8$, Lemma 4.10 gives a length $2^t\le n$, width 2, alphabet size *d* permutation branching program *B* and a family of auxiliary graphs ${\mathcal {H}}=(H_i,\ldots ,H_{i+t-1})$ satisfying the constraint such that ${{\,\textrm{INW}\,}}_{\mathcal {H}}$ fails to (1/20)-fool *B*, so it remains to show how to embed this into a length-*n* construction.

Note that for $j\in \{i,\ldots ,i+t-1\}$, we have $|H_j|= \prod _{k=i - 1}^{j-1}d_k$ where $d_{i - 1} = d$ and $d_k=\deg (H_k)$. Let us define a new family of graphs $(H^{\prime }_1, \dots , H^{\prime }_{\ell })$. For $j < i$, let $H_j^{\prime } = J_*$.

Observe that $H_{i - 1}^{\prime }$ is a graph on vertex set $[d]^{2^{i - 2}}$. Now we define $H_i^{\prime }$ as follows. The vertex space is $[d]^{2^{i - 1}}$, which we think of as $[d] \times [d]^{2^{i - 1} - 1}$. Let $(a_{i - 1}, b_{i - 1}) \in [d] \times [d]^{2^{i - 1} - 1}$ be a vertex, and let $(a_i, b_i)$ be an edge label, where $a_i \in [d_i]$ and $b_i \in [d]^{2^{i - 1} - 1}$. Then we let

$$\begin{aligned} H_i^{\prime }[(a_{i - 1}, b_{i - 1}),(a_i,b_i)] = (H_i[a_{i - 1}, a_i], J_*[b_{i - 1}, b_i]). \end{aligned}$$

More generally, for $j \in \{i, \dots , i + t - 1\}$, we define $H^{\prime }_j$ as follows. Let $(a_{i - 1}, b_{i - 1}, \dots , a_{j - 1}, b_{j - 1})$ be a vertex and let $(a_j, b_j)$ be an edge label, where $a_k \in [d_k]$ and $b_k \in [d]^{(2^{i - 1} - 1) \cdot 2^{k - i}}$ for each $k \in \{i - 1, \dots , j - 1\}$. Then

$$\begin{aligned} H^{\prime }_j[(a_{i - 1}, b_{i - 1}, \dots , a_{j - 1}, b_{j - 1}), (a_j, b_j)] = (a^{\prime }_{i - 1}, b^{\prime }_{i - 1}, \dots , a^{\prime }_{j - 1}, b^{\prime }_{j - 1}), \end{aligned}$$

where $(a^{\prime }_{i - 1}, \dots , a^{\prime }_{j - 1}) = H_j[(a_{i - 1}, \dots , a_{j - 1}), a_j]$ and $(b^{\prime }_{i - 1}, \dots , b^{\prime }_{j - 1}) = J_*[(b_{i - 1}, \dots , b_{j - 1}), b_j]$. Note that with this definition, $H^{\prime }_j$ is isomorphic as a graph to $H_j \otimes J_*$, and hence $\lambda (H_j^{\prime })\le \lambda (H_j)$ by Proposition 2.9. Finally, for $j \ge t$, let $H^{\prime }_j = J_*$.

The new program $B^{\prime }$ will ignore the final $n-2^t$ layers. Since the first $i-1$ graphs in the family ${\mathcal {H}}^{\prime }$ are $J_d, J_{d^2}, \ldots ,J_{d^{2^{i-2}}}$, we modify *B* such that it only reads the *first* symbol of each block of length $2^{i - 1}$, with identity transitions on all other symbols. Thus, the function computed by $B^{\prime }$ is given by

$$\begin{aligned} B^{\prime }(\sigma _1, \dots , \sigma _n) = B(\sigma _1, \sigma _{1 + 2^{i-1}}, \sigma _{1 + 2^{i-1} \cdot 2}, \dots , \sigma _{1 + 2^{i-1} \cdot (2^t - 1)}). \end{aligned}$$

Then the distribution of outputs of ${{\,\textrm{INW}\,}}_{{\mathcal {H}}^{\prime }}$ on symbols $1,1+2^{i-1},\ldots ,1+2^{i-1}\cdot (2^t - 1)$ will be identical to that of ${{\,\textrm{INW}\,}}_{{\mathcal {H}}}$, so ${{\,\textrm{INW}\,}}_{{\mathcal {H}}^{\prime }}$ will fail to (1/20)-fool the alphabet size *d*, length *n* modified program. $\square$

Dividing $[\ell ]$ into at most $\ell /t+1\le 2\ell /t$ blocks of size at most *t*, we have by Claim 4.11 that $\sum _{i=1}^\ell \lambda _i < 16\ell /t$, so at least $\ell /2$ of the constraints satisfy $\lambda _i<32/t$. Let *I* be the indices such that this occurs. Then let

$$\begin{aligned} \gamma _i = {\left\{ \begin{array}{ll} 32/t &  i\in I\\ 1 &  \text {else}.\end{array}\right. } \end{aligned}$$

Then $s_{{{\,\textrm{INW}\,}}}(d,\lambda _1,\ldots ,\lambda _\ell )\ge s_{{{\,\textrm{INW}\,}}}(d,\gamma _1,\ldots ,\gamma _\ell )=\Omega (\log (t)\cdot (\log n-O(\log \log n)))=\Omega (\log n\cdot \log \log (\min (n,d))$ via Lemma 2.7. $\square$

### Fooling in Spectral Norm

We now formally state our lower bound for fooling programs in spectral norm (which we precisely define in Definition 4.14).

#### Theorem 4.12

(Formal Statement of Theorem 1.11) For every $n=2^\ell$ and $\lambda _1,\ldots ,\lambda _\ell \ge 0$, if ${{\,\textrm{INW}\,}}(2,\lambda _1,\ldots ,\lambda _\ell )$ 1/3-fools ordered permutation branching programs of length *n*, width *w*, and alphabet size 2 with respect to spectral norm, then $s_{{{\,\textrm{INW}\,}}}(2,\lambda _1,\ldots ,\lambda _\ell )=\Omega (\log n\cdot \log \log (\min \{n,w\}))$.

To prove this, we introduce notation for distributions over a branching program, using the notation of Reingold et al. [30].

#### Definition 4.13

Given a length *n*, width *w*, alphabet size *d* branching program *B* with transition functions $B_1,\dots ,B_n$, for $t\in [n]$ let ${{\textbf{B}}}_t:[d]\rightarrow \{0,1\}^{w\times w}$ be defined where ${{\textbf{B}}}_t[s]_{i,j} =1$ if $B_t(i,s)=j$ and 0 otherwise. For $0\le i<j\le n$ let ${{\textbf{B}}}_{i..j}$ be defined as ${{\textbf{B}}}_{i..j}[s_{i+1}\dots s_{j}]={{\textbf{B}}}_{i+1}[s_{i+1}]\cdots {{\textbf{B}}}_j[s_j]$, and let ${{\textbf{B}}}={{\textbf{B}}}_{0..n}$. For a function ${{\,\textrm{GEN}\,}}:[S]\rightarrow {[d]^n}$, define the *distribution of B* *on *${{\,\textrm{GEN}\,}}$ as $\overline{{{\textbf{B}}}\circ {{\,\textrm{GEN}\,}}}=\mathop {\mathrm {\mathbb {E}}}\limits [{{\textbf{B}}}[{{\,\textrm{GEN}\,}}(U_{[S]})]].$ Furthermore, we define $\overline{{{\textbf{B}}}\circ U_{[d]^n}}=\mathop {\mathrm {\mathbb {E}}}\limits [{{\textbf{B}}}[U_{[d]^n}]].$

Note that this definition exactly matches Definition 2.2 when the branching program is equal to $G^{(n)}$ for a consistently labeled graph *G* (with $B_t=G$ for all steps *t* as in Remark 4.2). We can then define fooling with respect to a norm.

#### Definition 4.14

Let $\Vert \cdot \Vert$ be a norm on $w\times w$ real matrices and ${\mathcal {B}}$ a set of ordered branching programs of length *n*, width *w*, and alphabet size *d*. We say a function ${{\,\textrm{GEN}\,}}:\{0,1\}^s\rightarrow {[d]^n}$ $\varepsilon$-*fools *${\mathcal {B}}$ *with respect to* $\Vert \cdot \Vert$ if for every $B\in {\mathcal {B}}$ we have

$$\begin{aligned} \left\| \overline{{{\textbf{B}}}\circ {{\,\textrm{GEN}\,}}}-\overline{{{\textbf{B}}}\circ U_{[d]^n}}\right\| \le \varepsilon . \end{aligned}$$

To use this definition, we need to select a matrix norm. We define several different norms on matrices ${\textbf{A}}\in \mathbb {R}^{w\times w}$. Note that throughout the paper, all vectors are *row* vectors. Some examples include:

- $\Vert {\textbf{A}}\Vert _2 = \max _{x\in \mathbb {R}^w-\{0\}} \Vert x{\textbf{A}}\Vert _2/\Vert x\Vert _2$. We call this the *spectral norm*, and it is what we obtain bounds against.
- $\Vert {\textbf{A}}\Vert _{1} = \max _{x\in \mathbb {R}^w-\{0\}}\Vert x{\textbf{A}}\Vert _1/\Vert x\Vert _1 = \max _i \Vert {\textbf{A}}_{i,\cdot }\Vert _1$ where ${\textbf{A}}_{i,\cdot }$ is the *i*th row of ${\textbf{A}}$.
- $$\Vert {\textbf{A}}\Vert _{\max } = \max _{i,j}|{\textbf{A}}_{i,j}|$$.

We remark that fooling in $\ell _1$ norm is equivalent (up to a factor of 2) to the conventional notion of fooling programs with an arbitrary set of accept vertices, and fooling in $\max$-norm is equivalent to fooling programs with a *single* accept vertex. We work with $\ell _1$ norm in Appendix B, whereas here we obtain bounds against spectral norm.

We now prove the main lemma for spectral fooling of polynomial width permutation branching programs over a binary alphabet.

#### Lemma 4.15

For every $n=2^\ell$ and every constraint $(\lambda _1,\ldots ,\lambda _\ell )$ where $\sum _{i=1}^\ell \lambda _{i} \ge 8$, there is a family of auxiliary graphs ${\mathcal {H}}=(H_1,\ldots ,H_\ell )$ where $\lambda (H_i) \le \lambda _i$ and a length *n*, width $O(\sqrt{n})$, alphabet size 2 permutation branching program *B* such that the INW generator constructed with ${\mathcal {H}}$ fails to (1/3)-fool *B* with respect to spectral norm.

#### Proof

Our distinguishing permutation branching program is again a consistently labeled graph, with transitions equal at every layer, as in Remark 4.2. For every $m\in \mathbb {N}$, let $C_m$ be the 2-regular consistently labeled undirected *m*-cycle and let $v_2$ be a normalized eigenvector of $\overline{{\textbf{W}}}_{C_m}$ with second largest eigenvalue. For an $m\times m$ matrix ${\textbf{A}}$, let $\lambda _{2}({\textbf{A}}){\mathop {=}\limits ^{\text {def}}}v_2{\textbf{A}}v_2^T$, and recall that $\lambda _{2}(\overline{{\textbf{W}}}_{C_m})=\cos (2\pi /m)=1-\Theta (1/m^2)$ (see e.g. [27]). We will apply this definition to matrices ${\textbf{A}}\in \operatorname {span}\{{\textbf{I}},\overline{{\textbf{W}}}_{C_m},\overline{{\textbf{W}}}_{C_m}^2,\ldots \}$; $v_2$ is an eigenvector of all these matrices, but it is not always the second eigenvector. Nonetheless, it is convenient for us to measure expansion with respect to $v_2$. Note that when $v_2$ is an eigenvector of ${\textbf{A}}$, $\lambda _2({\textbf{A}}^k)=\lambda _2({\textbf{A}})^k$ for all *k*.

Recall that $\lambda _2(\overline{{\textbf{W}}}_{C_m}^n)=\lambda _2(\overline{{\textbf{W}}}_{C_m})^n=(1-\Theta (1/m^2))^n$. Now given *n*, choose $w=\Theta (\sqrt{n})$ to be some integer such that random walks of length *n* are 1/3 mixed with respect to $\lambda _2$, but walks of length *n*/2 are not. Formally let $w=\mathop {\mathrm {arg\,min}}\limits _{m \in \mathbb {N}}(1/9\le \lambda _{2}(\overline{{\textbf{W}}}_{C_m}^n)<1/3)$.

We then observe that $G=C_w$ is reversible, so we apply Lemma 4.5 with *G* and $(\lambda _1,\ldots ,\lambda _\ell )$ and obtain a PRG ${{\,\textrm{INW}\,}}_{\mathcal {H}}$ where ${\mathcal {H}}$ satisfies the constraint.

Intuitively, “wasting” a constant fraction of steps by not making progress on mixing is enough to distinguish INW output from truly random in spectral norm. Since $\lambda _{2}(\overline{{\textbf{W}}}_{C_w}^a)\le \lambda _{2}(\overline{{\textbf{W}}}_{C_w}^b)$ for all $a\ge b$, we again obtain a lower bound by Lemma 4.5:

$$\begin{aligned} \lambda _{2}(\overline{{\textbf{W}}_{C_w^n}\circ {{\,\textrm{INW}\,}}_{\mathcal {H}}})\ge \frac{1}{100}\lambda _{2}(\overline{{\textbf{W}}}_{C_w}^{n})+\frac{99}{100}\lambda _{2}(\overline{{\textbf{W}}}_{C_w}^{n/4}) = \frac{1}{100}\lambda _{2}(\overline{{\textbf{W}}}_{C_w})^{n}+\frac{99}{100}\lambda _{2}(\overline{{\textbf{W}}}_{C_w})^{n/4}. \end{aligned}$$

But then

$$\begin{aligned}&\left\| \overline{{\textbf{W}}_{C_w^n}\circ {{\,\textrm{INW}\,}}_{\mathcal {H}}}-\overline{{\textbf{W}}_{C_w^n}\circ U_{\{0,1\}^n}}]\right\| _2 \\&\quad \ge v_2(\overline{{\textbf{W}}_{C_w^n}\circ {{\,\textrm{INW}\,}}_{\mathcal {H}}}-\overline{{\textbf{W}}}_{C_w}^n)v_2^T\\&\quad = \lambda _{2}(\overline{{\textbf{W}}_{C_w^n}\circ {{\,\textrm{INW}\,}}_{\mathcal {H}}})-\lambda _{2}(\overline{{\textbf{W}}}_{C_w}^{n})\\&\quad \ge \frac{1}{100}\lambda _{2}(\overline{{\textbf{W}}}_{C_w})^{n}+\frac{99}{100}\lambda _{2}(\overline{{\textbf{W}}}_{C_w})^{n/4}-\lambda _{2}(\overline{{\textbf{W}}}_{C_w})^{n}\\&\quad \ge \frac{99}{100} \cdot \min _{x\in [1/9,1/3)}(x^{1/4}-x) > .42, \end{aligned}$$

where the final line follows from a numerical calculation, so we have the desired separation. $\square$

We can then use this lemma to prove Theorem 4.12.

#### Proof of Theorem 4.12

Let $t=\lfloor \log (\min \{n,w\})\rfloor$ and fix an arbitrary constraint $(\lambda _1,\ldots ,\lambda _\ell )$ such that ${{\,\textrm{INW}\,}}(d,\lambda _1,\ldots ,\lambda _\ell )$ (1/3)-fools the model with respect to spectral norm.

#### Claim 4.16

Every block $(\lambda _i,\ldots ,\lambda _{i+t-1})$ satisfies $\sum _{j=i}^{i+t-1}\lambda _j<8$.

The proof of the claim is essentially identical to that of Claim 4.11. Let us now use the claim to prove the theorem. Dividing $[\ell ]$ into at most $\ell /t+1\le 2\ell /t$ blocks of size at most *t*, we have by the claim that $\sum _{i=1}^\ell \lambda _i < 16\ell /t$, so at least $\ell /2$ of the constraints satisfy $\lambda _i<32/t$. Let *I* be the indices such that this occurs. Then let

$$\begin{aligned} \gamma _i = {\left\{ \begin{array}{ll} 32/t &  i\in I\\ 1 &  \text {else}.\end{array}\right. } \end{aligned}$$

Then $s_{{{\,\textrm{INW}\,}}}(d,\lambda _1,\ldots ,\lambda _\ell )\ge s_{{{\,\textrm{INW}\,}}}(d,\gamma _1,\ldots ,\gamma _\ell )=\Omega (\log (t)\cdot (\log n-O(\log \log n)))=\Omega (\log n\cdot \log \log (\min \{n,w\})$ via Lemma 2.7. $\square$

## Dependence on Width

In this section, we prove Theorem 1.8, establishing a lower bound on the seed length as a function of the width of the permutation branching program. Since we prove the INW generator does not even hit the distinguisher, we recall the formal definition of a hitting set generator.

### Definition 5.1

Let ${\mathcal {F}}$ be a class of functions $f: {[d]^n}\rightarrow \{0, 1\}$. An $\varepsilon$-*hitting set generator* ($\varepsilon$-*HSG*) for ${\mathcal {F}}$ is a function ${{\,\textrm{GEN}\,}}: \{0,1\}^s \rightarrow {[d]^n}$ such that for every $f \in {\mathcal {F}}$ where $\mathop {\mathrm {\mathbb {E}}}\limits _{x \leftarrow U_{{[d]^n}}}[f(x)]> \varepsilon$, there exists $y \in \{0,1\}^s$ such that $f({{\,\textrm{GEN}\,}}(y))=1$.

We are now prepared to give the formal statement.

### Theorem 5.2

(Formal Statement of Theorem 1.8) For every *d* and $n=2^\ell$ and $w\le 2^{n/2}$ and $\lambda _1,\ldots ,\lambda _\ell \ge 0$, if ${{\,\textrm{INW}\,}}(d,\lambda _1,\ldots ,\lambda _\ell )$ is a (1/2)-hitting set generator for ordered permutation branching programs (with arbitrary sets of accept vertices) of length *n*, width *w*, and alphabet size *d*, then $s_{{{\,\textrm{INW}\,}}}(d,\lambda _1,\ldots ,\lambda _\ell )=\Omega (\log (w) \cdot (\log (n)-\log \log (w))$.

The proof proceeds by showing that any INW PRG must constrain almost all spectral gaps to be at most $1/w^{\Omega (1)}$. To do this, we establish that if there is a constraint where $\lambda _r>1/w^{c}$ for some $c>0$, there is a graph *E* on $\sqrt{w}$ vertices and a permutation branching program that perfectly distinguishes between a pair of edges in *E* and random vertices. To enable this to work for all levels of the PRG, we tensor *E* with a large complete graph. We now state the main lemma.

### Lemma 5.3

There exists $c>0$ and $w_0 \in \mathbb {N}$ such that for all $d \in \mathbb {N}$ and $w\ge w_0$, for all $r\ge 1+\log \log w$, for every constraint $(\lambda _1,\ldots ,\lambda _r)$ such that $\lambda _r>1/w^c$, there is a family of auxiliary graphs ${\mathcal {H}}=(H_1,\ldots ,H_r)$ where $\lambda (H_i) \le \lambda _i$ and an alphabet size *d*, width $w^2$, length $2^r$ permutation branching program *B* such that $\Pr [B(U_{[d]^{2^r}})=1]\ge 1-w^{-\Omega (1)}$ and ${{\,\textrm{INW}\,}}_{r}$ fails to hit *B*.

To prove this, we recall the existence of expanders that are not too dense.

### Proposition 5.4

(see e.g. [31]) There are global constants $c>0$ and $v_0\in \mathbb {N}$ such that for every $S \ge v_0$, there is an undirected regular graph *Z* on *S* vertices such that $\deg (Z)<\sqrt{S}$ and $\lambda (Z)<1/S^c$.

We furthermore prove that we can approximately tensor these graphs, in effect creating block expanders on an arbitrary number of vertices:

### Proposition 5.5

There are global constants $c>0$ and $v_0\in \mathbb {N}$ such that for every $d\ge b\ge v_0$, there is a regular graph $Z = (V, E)$ on *d* vertices with the following properties.

- There is a partition $p:V\rightarrow [b]$ such that $V_i:=|p^{-1}(i)|\in \{\lfloor d/b\rfloor , \lceil d/b\rceil \}$ for every *i*.
- There is a regular graph *N* on [*b*] with degree $r\le \sqrt{b}$ such that if $(u, v) \in E$, then $(p(u), p(v)) \in E(N)$ or $p(u) = p(v)$.
- $$\lambda (Z)\le 1/b^c+b/d$$.

### Proof

For convenience, we will use positive rational *edge weights* in our definition of the graph *Z*. Such a graph can be converted into an unweighted graph by duplicating edges, similar to Definition 3.3.

Let *N* be the graph on *b* vertices of Proposition 5.4. Let $a:= \lfloor d/b \rfloor$. To define *Z*, we begin by blowing up each vertex $v_i$ in *N* to a cloud $V_i$ of size either *a* or $a + 1$, such that there are *d* vertices in total. Then, for every undirected edge $\{v_i, v_j\}$ in *N*:

- Add a complete bipartite graph between $V_i$ and $V_j$ to *Z* in which each edge has weight 1.
- If $|V_i| = a$, then add a clique to $V_j$, including self loops, in which each edge has weight $1/|V_j|$, thus increasing the weighted degree of each vertex in $V_j$ by one.
- Similarly, if $|V_j| = a$, then add a clique to $V_i$, including self loops, in which each edge has weight $1/|V_i|$.

By construction, every vertex in *Z* has weighted degree precisely $\deg (N) \cdot (a + 1)$, so *Z* is regular. Furthermore, at each vertex in *Z*, the weight of the incident “clique edges” is at most $\deg (N)$, i.e., a $(\frac{1}{a + 1})$-fraction of the total weight of all incident edges.

Now we show that *Z* has the claimed expansion. Let ${\widetilde{{\textbf{W}}}} \in \mathbb {R}^{d \times d}$ be the random walk matrix of *Z* and let ${\textbf{W}}\in \mathbb {R}^{b(a + 1) \times b(a + 1)}$ be the random walk matrix of the tensor product $N \otimes J_{a + 1}$. Let ${\textbf{P}}\in \mathbb {R}^{b(a + 1) \times d}$ be the “truncation matrix,” i.e., $x {\textbf{P}}$ consists of the first *d* entries of *x*, and let ${\textbf{L}}\in \mathbb {R}^{d \times b(a + 1)}$ be the “padding matrix,” i.e., $x {\textbf{L}}$ consists of *x* followed by $b(a + 1) - d$ zeroes. Then we can write

$$\begin{aligned} {\widetilde{{\textbf{W}}}} = {\textbf{L}}{\textbf{W}}{\textbf{P}}+ \mathbf {\Delta }, \end{aligned}$$

where the “error matrix” $\mathbf {\Delta } \in \mathbb {R}^{d \times d}$ is given by

$$\begin{aligned} (x \mathbf {\Delta })_i = w_{p(i)} \cdot \frac{1}{|p^{-1}(p(i))|} \cdot \sum _{j \in p^{-1}(p(i))} x_j, \end{aligned}$$

where $w_{p(i)} \in [0, \frac{1}{a + 1}]$ is the fraction of “clique edges” among all the edges incident to each vertex in the cloud $V_{p(i)}$. Consequently, for any test vector $x \in \mathbb {R}^d$ with $\Vert x\Vert _2 = 1$ and $x \perp \vec {1}$, we have

$$\begin{aligned}&\Vert x {\widetilde{{\textbf{W}}}}\Vert _2 \le \Vert x {\textbf{L}}{\textbf{W}}{\textbf{P}}\Vert _2 + \Vert x \mathbf {\Delta }\Vert _2 \\&\quad \le \lambda (N) + \sqrt{\sum _{j \in [b]} w_j^2 \cdot |p^{-1}(j)| \cdot \left( \frac{1}{|p^{-1}(j)|} \cdot \sum _{i \in p^{-1}(j)} x_i\right) ^2} \\&\quad \le \lambda (N) + \sqrt{\sum _{j \in [b]} w_j^2 \cdot |p^{-1}(j)| \cdot \frac{1}{|p^{-1}(j)|} \cdot \sum _{i \in p^{-1}(j)} x_i^2 } \\&\quad \le \lambda (N) + \frac{1}{a + 1}. \end{aligned}$$

$$\square $$

We note that counterintutively, our *lower bound* relies on the existence of expanders with an *upper bound* on their degree.

### Proof of Lemma 5.3

Let $l = \lfloor \log _d(w)\rfloor$. We break into cases depending on if *l* is strictly greater than 0 (equivalently, if $d\le w$ or $d>w$).

1. Case 1: $l>0$. Here, let *Z* be the graph on $S:=d^l \in [\sqrt{w},w]$ vertices with $\deg (Z)\le \sqrt{S}$ and $\lambda (Z)\le 1/w^c$ obtained from Proposition 5.4.
2. Case 2: $l=0$. Let *Z* be the *d*-vertex graph of Proposition 5.5 with $b=\lfloor w^{1/8} \rfloor$, and let *N* be the associated block graph. Observe that $\lambda (Z)\le 1/w^{c/8}+1/w^{7/8}\le 1/w^{c^{\prime }}$.

Now, in both cases let the graph family be ${\mathcal {H}}=(J_*,\ldots ,J_*,Z\otimes J_*)$ and observe that it satisfies the expansion constraint by Proposition 2.9. Next, we show that both cases can be distinguished from uniform output by a PRG, in both cases by checking if two blocks of symbols correspond to an edge in $H_r$.

1. Case 1: Let *B* be the permutation branching program that reads the symbols in coordinates $[1,l]\cup [2^{r - 1} + 1, 2^{r - 1} + l]$, and on reading (*x*, *y*), accepts if and only if $(x,y)\notin E(Z)$. This program has width $d^{2l}\le w^2$. We have by construction that ${{\,\textrm{INW}\,}}_{\mathcal {H}}$ does not hit *B*. Moreover, the probability that two random vertices in *Z* are not connected by an edge is at least $(S-\sqrt{S})/S\ge 1-w^{-1/4}$, and thus $\Pr [B(U_{[d]^{2^r}})=1]\ge 1-w^{-1/4}$, so we obtain the desired result.
2. Case 2: Recall that $V_1,\ldots ,V_b$ is the partition of [*d*] induced by the vertices in *Z*, and note that $b\le w^2$. Let *B* be the permutation branching program that reads the symbols at coordinates 1 and $2^{r-1}+1$, and on reading (*x*, *y*), accepts if and only if
  $$\begin{aligned} p(x) \ne p(y) \quad \text {and} \quad (p(x), p(y)) \notin E(N). \end{aligned}$$
  Note that this can be implemented by a permutation branching program of width $b^2\le w$, as the program only needs *b* distinct vertices to remember the necessary information about each symbol. We have by construction that ${{\,\textrm{INW}\,}}_{\mathcal {H}}$ does not hit *B*.
  We claim that on a random input $(x,y)\leftarrow U_{[d]}\times U_{[d]}$, the random variable (*p*(*x*), *p*(*y*)) is $\varepsilon$-close to uniform over $[b] \times [b]$, where $\varepsilon = b \cdot (b/d) \le w^{-3/4}$. To see this, note that for every $i\in [b]$, $\Pr [p(x)=i]\in (1/b-1/d,1/b+1/d)$ and thus for every $i,j \in [b]$,
  $$\begin{aligned}\Pr [p(x)=i \wedge p(y)=j] \in (1/b^2-2/bd,1/b^2+3/bd).\end{aligned}$$
  Thus, the total variation distance from uniform over (*i*, *j*) is at most $b^2(3/bd)=3b/d\le 3w^{-7/8}$.
  Thus,
  $$\begin{aligned} \Pr [B(U_{[d]^{2^r}})=1]&= \Pr _{(x,y)\leftarrow U_{[d]^2}}[(p(x),p(y)) \notin E(N) \wedge p(x)\ne p(y)]\\&\ge \Pr _{(u,v)\leftarrow U_{[b]^2}}[(u,v) \notin E(N) \wedge u\ne v]-3w^{-7/8}\\&\ge 1-\sqrt{b}/b-w^{-1/16}-3w^{-7/8}. \end{aligned}$$

Thus, in both cases we obtain that the generator fails to hit the program, and the program has expectation $1-w^{-\Omega (1)}$. $\square$

We now apply this lemma to prove the theorem.

### Proof of Theorem 5.2

Let ${{\,\textrm{INW}\,}}(d,\lambda _1,\ldots ,\lambda _\ell )$ be a (1/2)-HSG for width *w*, length *n* branching programs. Let $t=\lfloor \log \log (\sqrt{w})\rfloor$ and note $t\le \ell$ by assumption on *w*. Now fix a family of graphs ${\mathcal {H}}=(H_1,\ldots ,H_\ell )$ satisfying this constraint and let $d_i=\deg (H_i)$ and $p_i=\prod _{j\le i}d_i$.

### Claim 5.6

For $i\le t$, we have $\log (p_i)=\Omega (2^i)$.

### Proof

Let $i_0$ be a constant, large enough that $2^{2^{i_0 - 1}}$ is larger than the constant $w_0$ in Lemma 5.3, and large enough that for all $w \ge 2^{2^{i_0 - 1}}$, the quantity $1 - w^{-\Omega (1)}$ that appears in Lemma 5.3 is bigger than 1/2. We will show by induction that for all $i_0 < i \le t$, we have

3 $$\begin{aligned} \log (p_i) \ge \alpha \cdot 2^i + 1, \end{aligned}$$

where $\alpha \in (0, 1)$ is a sufficiently small positive constant.

Let $i_0 < i \le t$. By Lemma 5.3 with $w = 2^{2^{i - 1}}$, we have $\lambda _i \le 2^{-c \cdot 2^{i - 1}}$ for some positive constant *c*. By Proposition 2.6, we have $d_i \ge \min \{2 / \lambda _i^2, (p_{i - 1} + 1) / 2\}$. We split into two cases depending on which term of the $\min$ is smaller.

First, suppose $d_i \ge 2/\lambda _i^2$. Then $\log (p_i) \ge \log (d_i) \ge c \cdot 2^i + 1$, so provided we choose $\alpha \le c$, (3) is satisfied. Now, suppose instead that $d_i \ge (p_{i - 1} + 1) / 2$. If $i > i_0 + 1$, then $\log (p_i) \ge 2 \log (p_{i - 1}) - 1$, so we are done by induction. Finally, suppose $i = i_0 + 1$ (the base case). Trivially, $p_{i - 1} \ge 2$, so $d_i \ge 3/2$, which implies $d_i \ge 2$ since $d_i$ is an integer, and therefore $p_i = p_{i - 1} \cdot d_i \ge 4$. Therefore, by choosing $\alpha < 2^{-i_0}$, we ensure that (3) holds in this case as well. $\square$

Now, for every $i > t$, we can apply Lemma 5.3 again (assuming *w* is sufficiently large) to get $\lambda _i \le 1/w^c$. Consequently, by Proposition 2.6, we have $d_i \ge \min \{2w^{2c}, p_t / 2\}$ and hence $\log (d_i) = \Omega (\log (w))$. Thus, we obtain a seed length lower bound of $\Omega (\log (w)(\log (n)-\log \log (w)))$. $\square$

## Appendix A General Branching Programs

In this appendix, we show that Theorem 5.2 can be strengthened for general (e.g. non-regular) ordered branching programs, in the sense that the hitting parameter becomes exponentially close to 1. Moreover, we recover the result of Brody and Verbin that spectral analyses of the INW generator require seed length $\Omega (\log ^2 n)$ even for width-3 BPs.

### Theorem A.1

For every $n=2^\ell$ and $\lambda _1,\ldots ,\lambda _\ell \ge 0$, there is a parameter $\varepsilon = 1-2^{-\Omega (n^{1/4})}$ such that if ${{\,\textrm{INW}\,}}(2,\lambda _1,\ldots ,\lambda _\ell )$ is an $\varepsilon$-HSG against ordered branching programs of length *n*, width 3, and alphabet size 2, then $s_{{{\,\textrm{INW}\,}}}(2,\lambda _1,\ldots ,\lambda _\ell )=\Omega (\log ^2 n)$.

To do this, we show that width 3 ordered branching programs can distinguish graphs with a polynomially small spectral gap.

### Lemma A.2

There exists $n_0,c>0$ such that for all $n=2^\ell \ge n_0$, for every constraint $(\lambda _1,\ldots ,\lambda _\ell )$ where there exists $\ell /4\ge r\ge \log \log n$ such that $\lambda _r>1/n^c$, there is a family of auxiliary graphs ${\mathcal {H}}=(H_1,\ldots ,H_\ell )$ where $\lambda (H_i) \le \lambda _i$ and a length *n*, width 3, alphabet size 2 branching program *B* such that $\Pr [B(U_{\{0,1\}^n})=1]\ge 1-2^{-n^{1/4}}$ and ${{\,\textrm{INW}\,}}_{\mathcal {H}}$ fails to hit *B*.

The proof is similar to the (sketched) proof in [14] that ordered branching programs can distinguish coins slightly biased towards 1, and is essentially the argument of Brody and Verbin [9].

### Proof

Let $2^s=S$ be the power of two satisfying $n^{1/8} \le S < n^{1/4}$. Let *E* be the graph on *S* vertices with $\deg (E)=D$ where $D<\sqrt{S}$ and $\lambda (E)\le 1/n^c$ obtained from Proposition 5.4, where we assume $S\ge v_0$ by our choice of $n_0$. Then for some $K=2^k \ge S$ to be chosen later define

$$\begin{aligned} H_r=E\otimes J_{K/S}. \end{aligned}$$

Given $v \in [K]$ and $(i,j)\in [D]\times [K/S]$, the neighbor relation of $H_r$ decomposes as:

$$\begin{aligned} H_r[v,(i,j)]=(E[v_s,i],J[v^{\prime },j]) \end{aligned}$$

where $v_s$ denotes the *s* bit prefix of *v* and $v^{\prime }$ the $k-s$ bit suffix. Choose $K=2^{2^{r-1}}$ and let the family be ${\mathcal {H}}=(J_2,\ldots ,J_{2^{2^{r-1}}},H_{r},J_*,\ldots ,J_*)$. Verifying that ${\mathcal {H}}$ satisfies the constraint is direct.

Then choose some $(x,y) \in [S]\times [S]$ such that $(x,y)\notin E$ and create the length *n*, width 3 branching program *B* computing the function $f:\{0,1\}^n\rightarrow \{0,1\}$ defined as

$$\begin{aligned} f(\sigma )=\bigvee _{i=0}^{n/2^r-1}(\sigma _{2^r\cdot i+1\dots 2^r\cdot i+s} = x \wedge \sigma _{2^r\cdot i+k+1\dots 2^r\cdot i+k+s} = y). \end{aligned}$$

Since (*x*, *y*) is chosen such that it will never be output by ${{\,\textrm{INW}\,}}_{\mathcal {H}}$ in the bits of output corresponding to neighbor relations in *E*, and these bits are precisely those checked by *B*, we have that ${{\,\textrm{INW}\,}}_{\mathcal {H}}$ fails to hit *B*. However, a truly random input satisfies each clause with probability $2^{-2s}\ge 2^{-\log (n)/2}$ and since there are $n/2^r\ge n^{3/4}$ such clauses, the accept probability is at least

$$\begin{aligned} \Pr [B(U_{\{0,1\}^n})=1]=1-(1-1/\sqrt{n})^{n^{3/4}}\ge 1-\exp (-n^{1/4}), \end{aligned}$$

so we obtain a super-constant HSG separation. $\square$

We can then prove the theorem:

### Proof of Theorem A.1

Applying Lemma A.2, every family ${{\,\textrm{INW}\,}}(2,\lambda _1,\ldots ,\lambda _\ell )$ that $1-\exp (-n^{1/4})$-fools ordered branching programs of length *n*, width 3 and alphabet size 2 must have $\lambda _i<1/n^{\Omega (1)}$ for all $i \in [\log \log n,\ldots ,\ell /4]$, so we obtain $s_{{{\,\textrm{INW}\,}}}(2,\lambda _1,\ldots ,\lambda _\ell )=\Omega (\log ^2 n)$ via Lemma 2.7. $\square$

## Appendix B Existence of Optimal INW Generators

Despite spectral analysis of INW PRGs already reaching lower bounds in multiple cases, and clearly being incapable of giving a full derandomization of space bounded computation, there *exists* an INW PRG with seed length matching that of the probabilistic method, for any class of functions:

### Theorem B.1

Let $n = 2^{\ell } \in \mathbb {N}$, let $d \in \mathbb {N}$, and let ${\mathcal {F}}$ be a family of functions $f :[d]^n \rightarrow \{0,1\}$. There is a family of graphs ${\mathcal {H}}=(H_1,\ldots ,H_\ell )$ such that ${{\,\textrm{INW}\,}}_{\mathcal {H}}$ $\varepsilon$-fools ${\mathcal {F}}$, and ${{\,\textrm{INW}\,}}_{\mathcal {H}}$ has seed length $O(\log \log |{\mathcal {F}}|+\log (n/\varepsilon )+\log \log (d))$.

In particular, there is an instantiation of the INW PRG with optimal seed length for ordered branching programs:

### Corollary B.2

For all $d,w,n,\varepsilon$, there is a family of graphs ${\mathcal {H}}$ such that ${{\,\textrm{INW}\,}}_{\mathcal {H}}$ $\varepsilon$-fools ordered branching programs of length *n*, width *w* and alphabet size *d*, and this generator has seed length $O(\log (nwd/\varepsilon ))$.

The proof of Corollary B.2 is slightly more involved than the standard proof that a random function is a good PRG with high probability, but it uses essentially the same idea. For the first $O(\log \log (nwd/\varepsilon ))$ levels, we use complete graphs to construct the generator, i.e., the generator is the trivial “identity PRG” on $O(\log (nwd/\varepsilon ))$ bits. At higher levels, we use a randomly-chosen *one-outregular* digraph, and argue that with high probability the INW generator constructed with this graph is a good approximation of the concatenation of two lower-level generators over every function in the family.

### Remark B.3

A digraph *H* of degree 1 on $2^s$ vertices can also be viewed as a *hash function* $h :\{0, 1\}^s \rightarrow \{0, 1\}^s$. Thus, our PRG has the form

$$\begin{aligned} G(x) = (x, h_1(x), h_2(x), h_1(h_2(x)), h_3(x), h_1(h_3(x)), \dots ), \end{aligned}$$

where $|x| = O(\log (nwd/\varepsilon ))$ and each hash function $h_i$ is “hard-coded” into the PRG. This PRG *G* is identical to Nisan’s PRG [8], except that in Nisan’s (explicit) construction, the hash functions $h_i$ are selected pairwise-independently by the PRG “at runtime,” requiring a longer seed.

Besides the similarity between the two constructions, there are also similarities between our analysis and Nisan’s analysis [8]. Like us, Nisan shows that after fixing several hash functions, a random choice for the next hash function is “good” with high probability. However, Nisan only needs to ensure that the hash function is “good” for *one* branching program, whereas we must establish “goodness” for all functions in ${\mathcal {F}}$ simultaneously.

We first prove a lemma on the existence of good graphs for all sufficiently high levels of the INW generator. We view the previous levels of the PRG simply as a fixed function with sufficient seed length, and use the probabilistic method to find a one-outregular digraph such that the INW generator constructed with this graph (and the fixed function as a base) approximates the concatenation of two copies of the fixed function.

### Lemma B.4

Let $d, m \in \mathbb {N}$, let $\varepsilon > 0$, let ${\mathcal {F}}$ be a family of functions $f :[d]^{2\,m} \rightarrow \{0, 1\}$, and let $G :[S] \rightarrow [d]^m$ where $S > \frac{1}{2} \cdot \varepsilon ^{-2} \cdot \ln (2|{\mathcal {F}}|)$. There exists a 1-outregular digraph *H* on *S* vertices such that if we define $G^{\prime }(x) = (G(x), G(H[x, 0]))$ and $(G, G)(x, y) = (G(x), G(y))$, then for every $f \in {\mathcal {F}}$, we have

$$\begin{aligned} \left| \overline{f\circ G^{\prime }} - \overline{f \circ (G,G)}\right| \le \varepsilon , \end{aligned}$$

where the notation ${\overline{g}}$ denotes the expected value of the function *g* under a uniform random input.

### Proof

Sample *H* uniformly at random from all 1-outregular graphs on *S* vertices, i.e., for each vertex $s\in [S]$ we include an edge (*s*, *z*) where $z\leftarrow U_{[S]}$.

Now fix an arbitrary $f\in {\mathcal {F}}$ and let $\alpha :=\overline{f\circ (G,G)}$. Let

$$\begin{aligned}X_s=f(G(s),G(H[s,0])) \end{aligned}$$

and thus $$\mathop {\mathrm {\mathbb {E}}}\limits _s[X_s] = \overline{f\circ G^{\prime }}$$.

Furthermore, we have that $X_s \in \{0,1\}$, and the $X_s$ are independent for all *s*. Using that the neighbor of every vertex *s* is distributed uniformly over the set of vertices:

$$\begin{aligned} \mathop {\mathrm {\mathbb {E}}}\limits _H\left[ \mathop {\mathrm {\mathbb {E}}}\limits _{s}[X_s]\right]&= \mathop {\mathrm {\mathbb {E}}}\limits _s\left[ \mathop {\mathrm {\mathbb {E}}}\limits _H[X_s]\right] \\&= \mathop {\mathrm {\mathbb {E}}}\limits _s[f(G(s),G(U_{[S]})))]\\&= \mathop {\mathrm {\mathbb {E}}}\limits _{s,s^{\prime }}f((G(s),G(s^{\prime })))\\&= \overline{f\circ (G,G)} =\alpha . \end{aligned}$$

We now apply the probabilistic method to show there exists such a good *H*. By Hoeffding’s inequality:

$$\begin{aligned}\Pr _{H}\left[ \left| \mathop {\mathrm {\mathbb {E}}}\limits _s[X_s] -\alpha \right| > \varepsilon \right] \le 2\exp (-2\varepsilon ^2S).\end{aligned}$$

Note that the failure probability is exponentially small in *S*, i.e. the number of seeds at the prior level, despite *H* having degree 1. Then for a random *H*, the probability $\mathop {\mathrm {\mathbb {E}}}\limits _s[X_s]$ fails to $\varepsilon$-approximate $\alpha$ for *any* $f\in {\mathcal {F}}$ is bounded by $2|{\mathcal {F}}|\exp (-2\varepsilon ^2S)$. Using the assumption $S > \frac{1}{2} \cdot \varepsilon ^{-2} \cdot \ln (2|{\mathcal {F}}|)$, we obtain that the failure probability is strictly below 1, so there is a graph *H* that is good for every $f\in {\mathcal {F}}$, and letting $G^{\prime }(x)=(G(x),G(H[x,0]))$ completes the proof. $\square$

We can then use this to prove the main theorem.

### Proof of Theorem B.1

Let ${\mathcal {F}}_i$ be the set of restrictions of functions in ${\mathcal {F}}$ to *i* (arbitrary) input variables. Observe that $|{\mathcal {F}}_i|\le |{\mathcal {F}}|(d+1)^n$, as every variable can be either assigned a value or left unrestricted. Let $L = \frac{1}{2} \cdot \varepsilon ^{-2} \cdot \ln (2 |{\mathcal {F}}| \cdot (d + 1)^n)$ be the corresponding bound from Lemma B.4.

Let *r* be the smallest integer such that $2^{2^r} > L$. For $k \in \{1,\ldots ,r\}$ let $H_k = J_{2^{2^{k-1}}}$. Now let $r \le k \le \ell$. By induction, assume that we have constructed $H_1, \dots , H_k$. Let ${\mathcal {H}}_k = (H_1, \dots , H_k)$ and $G_k = {{\,\textrm{INW}\,}}_{{\mathcal {H}}_k}$. If $k < \ell$, then let $H_{k + 1}$ be the 1-outregular graph obtained from Lemma B.4 with ${\mathcal {F}}= {\mathcal {F}}_{2^{k + 1}}$ and $G = G_k$. We prove by induction that $G_k$ $(\varepsilon \cdot (2^k - 1))$-fools ${\mathcal {F}}_{2^k}$. For all $k<r$ this is trivial. Now fix an arbitrary $f\in {\mathcal {F}}_{2^{k+1}}$. Note that

$$\begin{aligned} \mathop {\mathrm {\mathbb {E}}}\limits [f(G_{k+1}(U))]&\le \mathop {\mathrm {\mathbb {E}}}\limits [f(G_k(U)||G_k(U))] + \varepsilon  &   (\text {Lemma}~B.4)\\&\le \mathop {\mathrm {\mathbb {E}}}\limits [f(U_{[d]^{2^k}}||G_k(U))] + \varepsilon +\varepsilon \cdot (2^k - 1)\\&\le \mathop {\mathrm {\mathbb {E}}}\limits [f] + \varepsilon +2\varepsilon \cdot (2^k - 1) \end{aligned}$$

where the second and third lines use the inductive hypothesis. A similar argument holds for the opposite direction, so we obtain the desired result. Then by taking $\varepsilon \leftarrow \varepsilon /n$, we conclude.

Moreover, the seed length of ${{\,\textrm{INW}\,}}_{\mathcal {H}}=G_{\ell }$ is $\log (L)=O(\log \log (|{\mathcal {F}}|)+\log (n/\varepsilon )+\log \log (d))$. $\square$

## Appendix C Seed Length Lower Bound

For completeness, we include a proof of the seed length lower bound for *general* pseudorandom generators (not necessarily following the INW template) against permutation branching programs.

### Proposition C.1

Given $n,d\in \mathbb {N}$ and $\varepsilon >0$, let $G:\{0,1\}^s\rightarrow {[d]^n}$ be an $\varepsilon$-PRG for permutation branching programs of length *n*, width 2, and alphabet size *d*. Then $s=\Omega (\log (nd/\varepsilon ))$, provided $2^{-n}\le \varepsilon < 1/3$. Furthermore, the same lower bound holds if *G* is an $\varepsilon$-PRG for permutation branching programs with respect to spectral norm.

### Proof

First, note that for any matrix $M\in \mathbb {R}^{w\times w}$, we have $\Vert M\Vert _{\max }\le \Vert M\Vert _2$. From this, we obtain that lower bounds against $\delta$-fooling a program with a single accept vertex imply the equivalent lower bounds against $\delta$-fooling in spectral norm, since

$$\begin{aligned}\delta \le \left( \overline{{{\textbf{B}}}\circ G}-\overline{{{\textbf{B}}}\circ U_n}\right) _{v_0,v_{\text {acc}}} \le \left\| \overline{{{\textbf{B}}}\circ G}-\overline{{{\textbf{B}}}\circ U_n}\right\| _2.\end{aligned}$$

For the bounds against *n* and $\varepsilon$, let $v:[d]\rightarrow \{0,1\}$ be an approximately balanced function, i.e., $\mathop {\mathrm {\mathbb {E}}}\limits _{\sigma }[v(\sigma )] \in [1/3, 2/3]$.

1. First, we show that $s \ge \log (d)$. If $s\le \log (d)-1$, there are at most *d*/2 distinct first symbols output by *G*. Then there exists a width-2 permutation branching program *B* with a single accept vertex that accepts input $\sigma$ if and only if $\sigma _1$ is not output by *G*. This program satisfies $\Pr [B(U_{{[d]^n}})=1]\ge 1/2$ but $\Pr [B(G(U_s))=1]=0$ by construction, a contradiction.
2. Next, we show that $s \ge \Omega (\log (1/\varepsilon ))$. For $x\in {[d]^n}$ and $a\in \{0,1\}^n$ let . Set $l = \lfloor \log (1/\varepsilon ) / \log (3) \rfloor - 1$. For every $x\in \{0,1\}^l$, we have that
  is computable by an alphabet size *d*, width 2 permutation branching program, and hence *G* fools $g_a$ with error $\varepsilon$. By the well known AND trick [19], *G* thus fools
  for every *a* up to error $2\varepsilon$. As $\mathop {\mathrm {\mathbb {E}}}\limits [f_a(U_{{[d]^n}})]\ge 3^{-l}>2\varepsilon$, *G* hits every such program. But there are $2^l = (1/\varepsilon )^{\Omega (1)}$ such programs and each string output by *G* can hit at most one, so we conclude.
3. Finally, we show that $s \ge \log n$. For this step, we recall the proof of [18]:
  If $2^s \le n-1$, there is some nonzero vector $z \in \mathbb {F}_2^n$ such that for every *x*,
  $$\begin{aligned} \sum _{i = 1}^n z_i \cdot v(G(x)_i) \equiv 0 \pmod {2}. \end{aligned}$$
  The function $B(x) = \sum _{i = 1}^n z_i \cdot v(x_i) \bmod 2$ can be computed by a width-2 alphabet size-*d* permutation branching program with a single accept vertex, and $\Pr [B(U_{{[d]^n}}) = 1] \ge 1/3$, but $\Pr [B(G(U_s))=1]=0$, a contradiction.

$$\square $$

Finally, we note that by allowing an arbitrary set of accept vertices, we also obtain a lower bound in terms of width:

### Proposition C.2

Given $n,w,d\in \mathbb {N}$, if $G:\{0,1\}^s\rightarrow [d]^n$ is a (1/3)-PRG for permutation branching programs of width *w* with an arbitrary set of accept vertices then $s=\Omega (\log w)$, provided $w\le d^n$.

### Proof

If $w < d$, then we are done by the prior $\Omega (\log d)$ lower bound. Now assume $w \ge d$, and let $l = \lfloor \log _d(w) \rfloor \ge 1$. Our assumption $w \le d^n$ implies that $l \le n$, so we can define

$$\begin{aligned} S = \{(G(x)_1, \dots , G(x)_l) \in [d]^l: x \in \{0, 1\}^s\}. \end{aligned}$$

Assume for the sake of contradiction that $s \le l \log (d) - 1$. Then $|S| \le 2^s \le d^l / 2$. Let $B :[d]^n \rightarrow \{0, 1\}$ be the function

$$\begin{aligned} B(\sigma ) = 1 \iff (\sigma _1, \dots , \sigma _l) \notin S. \end{aligned}$$

The function *B* can be computed by a width *w*, length *n*, alphabet size *d* permutation branching program: the program stores the first *l* symbols of its input in its state, and then in the final layer, sequences in *S* are marked as rejecting and sequences outside *S* are marked as accepting. (Here we are using $d^l \le w$.) However, $\Pr [B(U_{{[d]^n}})=1]\ge 1/2$ and $\Pr [B(G(U_s))=1]=0$, a contradiction. Therefore,

$$\begin{aligned} s> l \log d - 1 > \frac{1}{2} \cdot \log _d(w) \cdot \log d - 1 = \frac{1}{2} \log w - 1. \end{aligned}$$

$$\square $$

## Footnotes

1 Braverman et al. [14] analyze the INW generator constructed with *randomness extractors* [20], but the extractor parameters they use follow from spectral expansion properties of the underlying graphs [21].

2 We do not require the domain to be a power of two, to avoid complications with non-power of two $d_i$’s.

3 Technically, the “undirected” graphs in this paper are really *symmetric directed* graphs. That is, each “undirected edge” $\{u, v\}$ represents two directed edges, (*u*, *v*) and (*v*, *u*). The labels of these two directed edges need not be equal.
